# Country-specific optimization strategy for testing through contact tracing can help maintain a low reproduction number ($R_{0}$) during unlock

**DOI:** 10.1038/s41598-021-03846-z

**Published:** 2022-01-07

**Authors:** Uddipan Sarma, Bhaswar Ghosh

**Affiliations:** 1 Vantage Research, Sivasamy St, CIT Colony, Mylapore, Chennai, Tamil Nadu 600004 India; 2 Center for Computational Natural Sciences, International Institute of Information Technology, Hyderabad, 500032 India

**Keywords:** Epidemiology, Computational models

## Abstract

In response to the COVID19 pandemic, many countries have implemented lockdowns in multiple phases to ensure social distancing and quarantining of the infected subjects. Subsequent unlocks to reopen the economies started next waves of infection and imposed an extra burden on quarantine to keep the reproduction number ($R_{0}$) < 1. However, most countries could not effectively contain the infection spread, suggesting identification of the potential sources weakening the effect of lockdowns could help design better informed lockdown-unlock cycles in the future. Here, through building quantitative epidemic models and analyzing the metadata of 50 countries from across the continents we first found that the estimated value of $R_{0}$, adjusted w.r.t the distribution of medical facilities and virus clades correlates strongly with the testing rates in a country. Since the testing capacity of a country is limited by its medical resources, we investigated if a cost–benefit trade-off can be designed connecting testing rate and extent of unlocking. We present a strategy to optimize this trade-off in a country specific manner by providing a quantitative estimate of testing and quarantine rates required to allow different extents of unlocks while aiming to maintain $R_{0} < 1$. We further show that a small fraction of superspreaders can dramatically increase the number of infected individuals even during strict lockdowns by strengthening the positive feedback loop driving infection spread. Harnessing the benefit of optimized country-specific testing rates would critically require minimizing the movement of these superspreaders via strict social distancing norms, such that the positive feedback driven switch-like exponential spread phase of infection can be avoided/delayed.

## Introduction

Declaration of the coronavirus pandemic by WHO severely overhauled global economic and social endeavors^1^. In response to the initial upsurge of infection spread^3,4^, many European and East Asian countries were able to contain the first wave successfully by imposing strong mitigation measures through nationwide lock-down coupled with rigorous testing and quarantine strategies. However, even with strict lock-downs, many countries struggled to contain the growth of infection^2^. During the pre-vaccine phase^5^ isolation of the infected population following aggressive testing and ensuring strong social distancing were the two most widely accepted ways globally to contain the infection spread and reduce the fatalities^6,7^. Despite the recent launching of several vaccine programs worldwide, complete immunization can take several months to years to cover the complete cross-section of susceptible age groups in all countries, especially in the economically fragile countries. Also, there are indications that vaccination alone may not be sufficient to completely contain COVID19^8^; this inevitably rearticulates the importance of occasional lockdown and quarantine strategies in the immediate future.

However lockdowns, also had major financial implications in the livelihood of millions of people, especially (but not restricted to) in low and medium-income countries^9–12^, calling for immediate plans to open up the economic activities. The unlocking measures then subsequently led to an increase in infection rates which initiated the next wave of infection in several countries who were largely successful in containing their respective first waves. The lockdown-unlock cycles were not successful to the extent desired and there could be scope of improvement in the strategies of testing and quarantining to maintain the basic economic activities while minimizing the spread of infection at the same time. The number of testing conducted per day in a country is limited by the country's resource capacity^13^ but it is also observed that the testing capacity is steadily rising in most of the countries^14–16^. Intuitively, the allowed extent of unlocking in a country should be a function of testing capacity as the daily testing captures the trend (increase or decrease) of infections per day, but it is not yet well established how testing facilities and extent of unlocking can be connected quantitatively. In this study we show that a country-specific cost–benefit trade-off between testing rate and unlocking extent can facilitate devising quantitative guidelines for unlocking, while considering the maximum resource capacity for testing within a given country.

Here, firstly we built a dynamic epidemic model^17–20^ that captures the spread of COVID19 infection and calibrated the model to country-specific time series data^1^ for confirmed, recovered and dead populations. We did this for 50 different countries with various stages of infection. The model simulations and subsequent analysis suggests how the extent of partial unlock (extent of unlock) and quarantine rates can be optimally combined to maintain $R_{0}$ < 1; this depends on transmission rate, quarantine capacity and associated cost of testing. We also connected the extent of unlock with the frequency of the periodic unlock^21^ time. Although the dynamic epidemic models build with ordinary differential equation (ODE) can quantitatively estimate infection spread parameters with high confidence they can not explicitly capture the relation between $R_{0}$ and testing rates, hence, we next developed an agent-based stochastic epidemic model which uses the country specific infection parameters optimized using the ODE model and then incorporates testing through contact tracing of individual infected agents. Using this hybrid modeling approach, we first estimated the testing rate required to maintain $R_{0} < 1$ and further studied how superspreaders^22^ can spread the infection during strict lockdowns; our simulations show that a small fraction of superspreaders can account for the majority of infections observed during the lockdowns which would then eventually be amplified further in the next cycle of unlocking. Indeed, most countries reported a continuous rise in infection during the lockdown, indicating such plausible underlying contribution from superspreaders. A recent study on two Indian states showed that a small fraction of superspreaders were responsible for transmitting ~80% of infection during lockdown^22^. Analysis of epidemic models specific to different countries shows a switch-like spread of infection can occur as a function of disease transmission rate and quarantine rate stemming from the implicit systems-level positive feedback loop primarily driving the spread of infection epidemics such as COVID19. Our analysis strongly suggests that minimizing the movement of superspreaders during the lockdowns can be very critical to the rapid success of such lockdowns and it can delay/circumvent the onset of the exponential increase in infection in the subsequent unlocks.

## Results

### Country specific doubling rate of the infection is dependent on the testing rate

**Figure 1:**
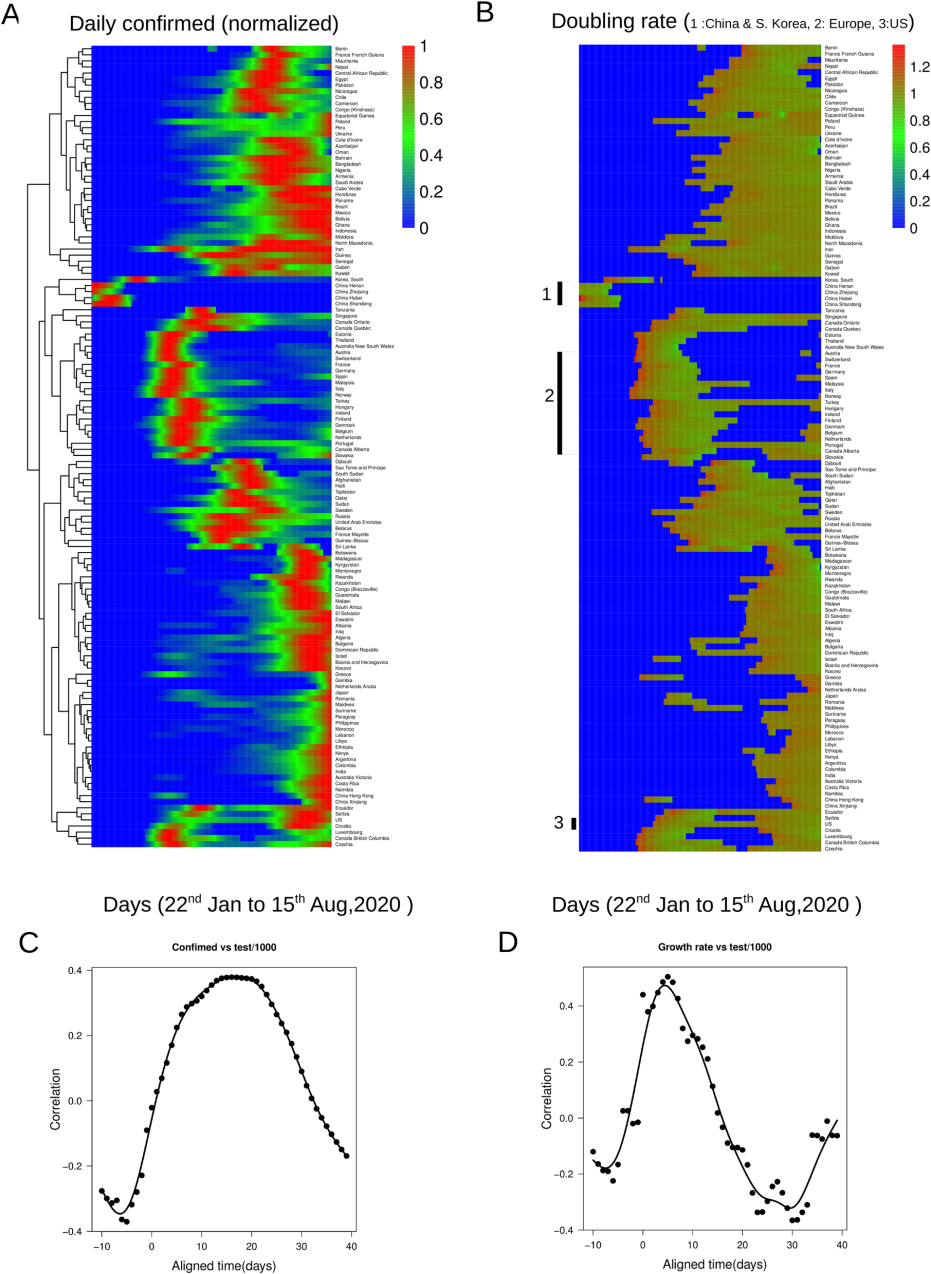
The Covid-19 outbreak in different countries and its relation with the testing rate. (**A**) The heat map displays the clustered dynamics for around 100 different countries for the daily cases normalized to the maximum for each country (Days correspond to 22nd January, 2020 and to 15th August, 2020). The dendrogram is based on hierarchical clustering of the time traces. The horizontal axis represents the time in days while the vertical axis corresponds to different countries. The color code provides the (normalized) number of confirmed cases. (**B**) The heat map represents the doubling rate as a function of time clustered according to the dynamics of the time traces as shown in (**A**). The horizontal axis represents the time in days while the vertical axis corresponds to different countries. The color code provides the doubling rates of confirmed cases. The results are shown on an exponential scale. (**C**) Pearson correlation coefficient of test rate with the daily confirmed cases at different time points along the dynamics are shown. The solid lines represent loess based local regression fits. The time traces are aligned w.r.t the maximum doubling rates. 0 on the x-axis corresponds to the maximum doubling rate for all the countries. (**D**) The Pearson correlation coefficient of test rate with the doubling rate at different time points along the dynamics aligned as described in (**C**). The solid lines represent loess based local regression fits.

To investigate the impact of testing on the epidemic dynamics in different countries, we first took the daily confirmed infection time course data from WHO^1^ and clustered the region-wise data according to their dynamic patterns. A hierarchical clustering algorithm (using pheatmap package in R) is used to analyze the dynamics of selected countries (countries with at least 1000 infections per day in their maximum infection spread phase between 15th Jan to 15th August 2020 were selected). Figure 1A shows provinces in China and S. Korea, for instance, are clustered together, as the infection spread there at the earliest times. Subsequently, infection in different parts of Europe/US is followed by infection in other Asian and South American countries (in Fig. 1A the color bar represents daily confirmed cases normalized to the maximum for each country (see Figure S1A for an enlarged version representing certain regions in the heatmap). Next, we calculated the doubling rate (the inverse of the doubling time which shows how much time does the population take to double the number of infections in each country) from the time series data of these countries, as shown in Fig. 1B. Both Fig. 1A, B comparatively show that infection peaks and doubling rates vary across countries. For a more direct comparison of the time evolution of the infection trajectories, we superimposed them. In order to superimpose the trajectories, the doubling rate time courses for the different countries were aligned at the maximum doubling rate (Details in SI). Linear correlations analysis (Methods) between different time points of the aligned trajectories and numbers of tests/10,000 population across these countries are shown in Fig. 1C. We observed that the normalized daily cases showed a positive correlation with the test rate around a time point of 16 days on the aligned time axis (Figs. 1C, S1C, where 0 corresponds to the time of maximum doubling rate), suggesting, countries with higher test rates tend to have higher confirmed cases. On the other hand, the doubling rate after around 26 days displays a negative correlation with test rate (Figs. 1D, S1B lower panel), hence, countries with higher testing rates are also able to reduce the rate of infection spread faster.

During the start of the infection, the doubling rate is usually high for a higher testing rate as indicated by a positive correlation. Thus, a higher testing rate (Figure S1B top panel) during the onset of infection allows more accurate estimation of the spread of infection which can be critical in containment of the infection at its early stages through preventive measures such as quarantine. Next, in order to quantitatively investigate the effect of testing rate and lockdown on $R_{0}$ we next took recourse to a quantitative epidemic model and subsequently an agent-based model.

### $R_{0}$ values during lockdown period exhibit significant dependency on the test rate

**Figure 2:**
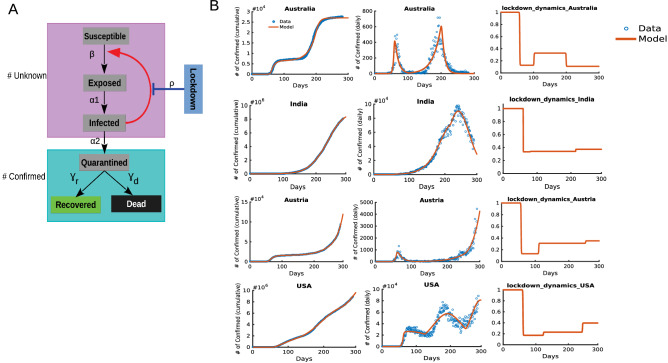
The description of the SEIQR model and calibration for different countries. (**A**) Schematic representation of the SEIQR model which contains susceptible, exposed, infected, quarantined, recovered and dead compartments. The lockdown is implemented through a sigmoid function as shown in the methods section. The quarantined, recovered and dead cases together comprise the confirmed cases. The arrow from infected to susceptible represents the positive feedback that fuels the infection spread in the population which in turn is negatively regulated by the lockdown, as indicated by a blunt headed bar. (**B**) The SEIQR models fit the data for 4 representative countries as indicated for the cumulative confirmed and daily confirmed cases. The number of days in X axis corresponds to the time course data available in JHU CSSE [41] where 0 corresponds to 22nd January, 2020 and the end time point corresponds to 30th October, 2020. The lockdown function in the third column shows the extent of lockdown (also the percentage of lockdown where 1 = 0% and 0 = 100% lockdown) which is also estimated by fitting during the model calibration.

To calculate the $R_{0}$ for different countries we next constructed a compartmental model to quantitatively capture the dynamics of infection. The model contained five compartments—susceptible (S), exposed (E), Infected (I), Quarantined (Q) and removed (R), where R contains both recovered (Re) and dead (D) population fractions^23^. Figure 2A shows the structure of the implemented SEIQR model. A susceptible person is exposed to the infection through transmission from an already infected person (rate parameter $\beta$). After exposure, the exposed individual (E) is infected too and moves to compartment I (rate parameter $\alpha_{1}$) and it subsequently gets quarantined in compartment Q (rate parameter $\alpha_{2}$). The quarantined individual eventually either recovers (rate parameter $\gamma_{r}$) or dies (rate parameter $\gamma_{d}$). In this model, we have not considered reinfection of the recovered individual. Infection dynamics were simulated by a set of 6 coupled ordinary differential equations. The SEIQR model thus constructed is next fitted to the confirmed (Co), recovered (Re) and dead (De) population trajectories obtained from the public data (details in methods and SI, section II).

Figure 2B shows the confirmed cumulative and daily cases, as well as the estimated lockdown extent (details of lockdown function in methods sections) and lockdown dynamics for four representative countries with different susceptible population sizes and stages of infection. Model fits for the 50 countries are shown in Supplementary Figure S2. The average incubation time independently estimated from the fitting infection dynamics of all the 50 countries (average of $\frac{1}{{\alpha_{1} }}$) is approximately 8.1 days which is close to the observed value^25^. Our simple SEIQR model thus quantitatively captured the cumulative infection dynamics of countries with different approaches towards lockdown and subsequent unlock implementation. Parametrizing the model with multiple country specific waves of infection (data until Oct 2020), where each wave is of distinct magnitude and duration (Fig. 2B, shown for four representative countries Australia, USA, Austria and India), resulted in country specific infection parameters of higher confidence. The model fitted to cumulative trajectories of a country also captured well the daily infection numbers in that country (Fig. 2B, 2nd column). We next used these country specific parameters to calculate the respective $R_{0}$ values.

**Figure 3:**
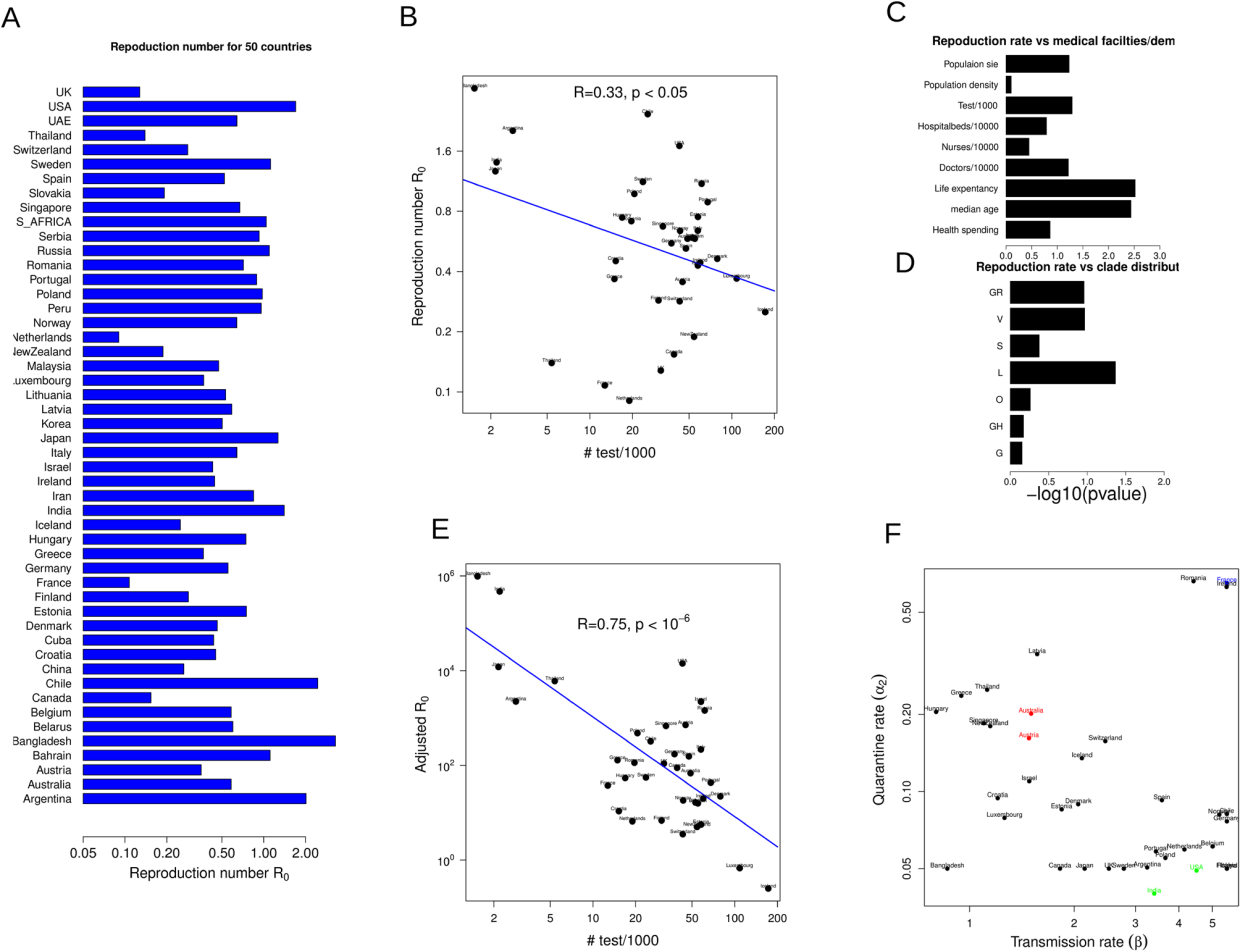
The reproduction number ($R_{0}$) displays correlation with the testing rate across countries. (**A**) The barplot shows $R_{0}$ for the fitted 50 countries after lockdown. (**B**) The scatter plot describes correlation between the test rate and $R_{0}$ after lockdown for the 39 countries with at least 10 complete genome sequences as of the end of June, 2020 described in the main text. (**C**) The statistical significances of the linear dependence of $R_{0}$ on different demographic and medical facilities factors are shown based on linear regression for the same 39 countries. (**D**) The statistical significance of linear dependence of $R_{0}$ on the frequencies of the clades (G, GR, GH, L, O, S, V) are shown. (**E**) The scatter plot indicates the correlation between the adjusted $R_{0}$ values and the test rates.$R_{0}$ values for each country are adjusted based on the correlation obtained in (**C**, **D**) described in the method section. (**F**) The values of the fitted transmission rates and quarantine rates for all the 50 countries are plotted and the representative countries for consideration of the cost–benefit analysis and agent based model simulation are shown in colors red, green and blue.

$R_{0}$ for different countries were calculated just after lockdown (Fig. 3A). The test rate displays a negative correlation with $R_{0}$ values (Fig. 3B), however, the correlation is not significant (correlation coefficient ~ 0.33). $R_{0}$ presumably depends on many other factors including demographics, medical facility and distribution of virus strains in the population which may interfere with the influence of test rate in spread of infection. In order to systematically adjust for the influence of such factors on $R_{0}$, we collected several publicly available datasets of demographics, medical facilities and genome sequences in a country specific manner^1^ (details in methods). $R_{0}$ values have a significant correlation with several of these factors (Figs. 3C, S3A). We also observed a statistically significant correlation of $R_{0}$ with the frequency of clades in the population; specifically the L and GR strains show significant influence on $R_{0}$(Figs. 3D, S3B). We next linearly adjusted the $R_{0}$ values (see methods) using the most significant factors derived from the correlation analysis-median age, population size, doctors/10,000 patient and frequency of the L clade-which shows that the adjusted $R_{0}$ values exhibit much higher correlation with the test rate (Fig. 3E. correlation coefficient 0.75). Here, for the correlation analysis, we choose countries only with at least 10 complete genome sequences till the end of June 2020 which selects 39 countries out of the original set of 50 countries.

Owing to the asymptomatic component of the COVID19 infection, testing the population is essential in order to quarantine the asymptomatic people. Thus, the quarantine rate would critically depend on the testing rate. Ideally a high testing rate would allow a country to unlock it’s economies and at the same time maintain a low infection rate. On the other hand, increasing the testing rate imposes extra financial and resource costs. To study the nature of this cost–benefit trade-off between quarantine and unlock, we specifically selected 5 representative countries with different transmission rates for further investigation: high transmission rates (USA, India), low transmission rates (Australia, Austria) and high transmission rate and high quarantine rate (France) (Fig. 3F shows countries with distinct transmission rates and quarantine rates).

### Optimum changes in country-specific quarantine rates can effectively reduce the infection post lockdown

Typically, when lockdown is removed, the transmission rate (β) is expected to increase. For our SEIQR model the $R_{0}$ value is given by $R_{0} = \frac{\beta }{{\alpha_{2} }}\frac{\rho }{{\rho_{max} }}$ (see SI section III for derivation) where $\rho$ captures the extent of lockdown ($\rho$ = 1 and $\rho = \rho_{max}$ represents full lockdown and full unlock respectively). From this expression, we can calculate the extent by which the quarantine rate ($\alpha_{2}$) must be increased in order to compensate for the effect of partial or full unlock such that R0 < 1 can be maintained during unlock. The fold change in $\alpha_{2}$ to keep the $R_{0}$ value close to one is given by (SI for derivation)

1 $$F_{\alpha } = \frac{\beta }{{\alpha_{2} }}\frac{\rho }{{\rho_{max} }}$$

**Figure 4:**
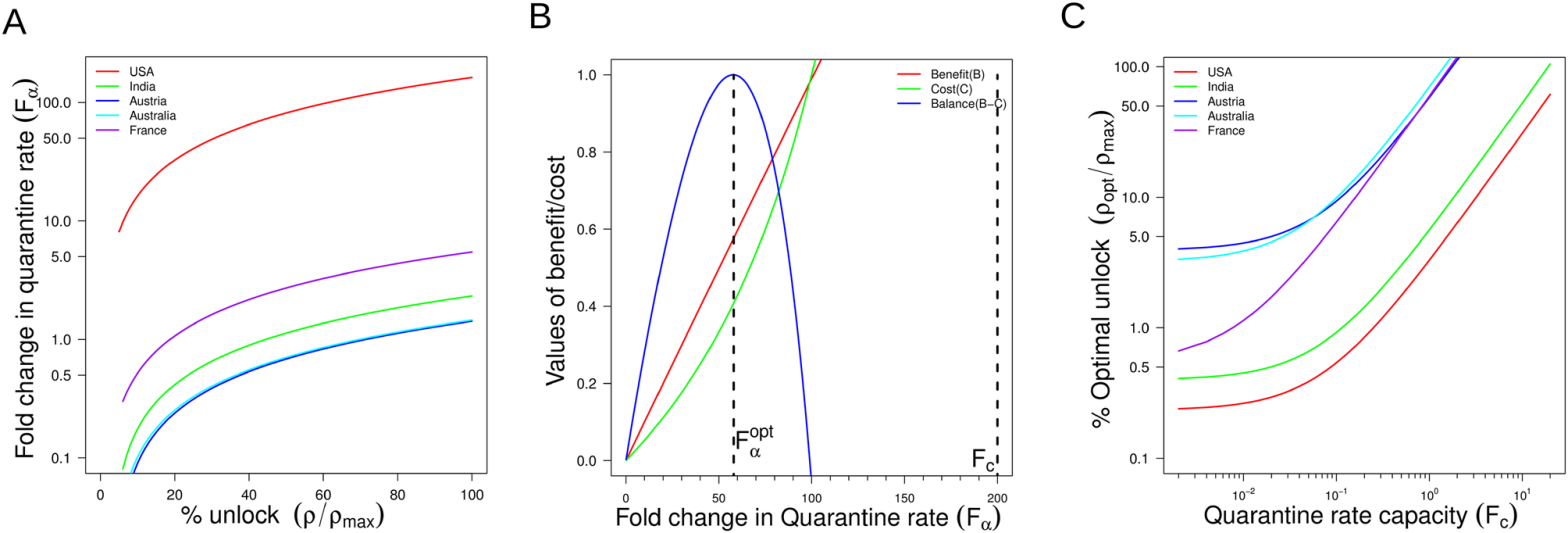
The optimization of trade-off between testing rate and extent of unlock. (**A**) The relationship between the extent of unlock and the corresponding quarantine rate required to keep $R_{0}$ values equal to one for five countries as indicated. The equation $F_{\alpha } = \frac{\beta }{{\alpha_{2} }}\frac{\rho }{{\rho_{max} }}$(SI IV) is plotted with $F_{\alpha }$ on y-axis and $\frac{\rho }{{\rho_{max} }}$ on x-axis for different country specific ($\alpha_{2} ,\beta$ values. The y-axis is plotted in log-scale. (**B**) The cost and benefit curves as a function of quarantine fold change for the countries. The benefit curve $B = \frac{{F_{\alpha } }}{{F_{0} + F_{\alpha } }}$(SI IV) is plotted at different values of $F_{\alpha }$ on x-axis for fixed value of $F_{0} = 10^{4}$. The cost curve C = $\lambda F_{c} \frac{{F_{\alpha } }}{{F_{c} - F_{\alpha } }}$(SI IV) is plotted at different values of $F_{\alpha }$ for fixed value of maximum quarantine capacity $F_{c} = 200$ and cost per quarantine capacity $\lambda = 5 \times 10^{ - 5}$. The cost–benefit is optimized at a value $F_{\alpha }^{opt}$ ~ 60 for a maximum capacity $F_{c}$ = 200 of the fold change in quarantine rate. (**C**) The optimum extent of unlock calculated from $F_{\alpha }^{opt}$ as a function of the maximum quarantine capacity $F_{c}^{{}}$ for different countries as indicated. The Eq. (3) for optimal unlock is plotted with $\frac{{\rho_{opt} }}{{\rho_{max} }}$ on the y-axis and $F_{c}$ on x-axis by fixing the parameters $F_{0} = 10^{4}$ and the cost per quarantine capacity , $\lambda = 5 \times 10^{ - 5}$ for different country specific ($\alpha_{2} ,\beta$ values as indicated. Both the axes are plotted in log-scale.

Here, $\rho = 1$ corresponds to a full lockdown case and a gradual increase in value of $\rho$ depicts partial unlock finally moving towards full unlock at $\rho = \rho_{max}$. The equation shows that the required fold change to compensate for the effect of lockdown removal would reduce as the extent of unlock reduces (Fig. 4A). In fact, our model fitting shows a higher transmission rate for the USA compared to Austria or Australia, asking for a higher quarantine rate in the USA for the same extent of unlock (Fig. 4A). However, for instance for France, in spite of a very high value of $\beta$, the required increase in quarantine is low due to its already high quarantine rate (Fig. 4A). Better control on infection spread during unlocks would require an increased quarantine rate which can only be achieved by rigorous contact tracing and testing. We studied this scenario as a cost–benefit tradeoff where on one hand removal of lockdown benefits economic activities but at the same time more testing needs to be conducted to circumvent the enhanced rate of infection spread. If we assume that the maximum quarantine capacity (of fold change) in $\alpha_{2}$ is $F_{c}$ and investment cost in testing is $\lambda$ per testing, the optimal fold change in $\alpha_{2}$ would be (see SI section IV for details)

2 $$F_{\alpha }^{opt} = F_{c} \frac{{\left( {\lambda \frac{{F_{c}^{2} }}{{F_{0} }}} \right)^{\frac{3}{2}} + 1}}{{\left( {\lambda \frac{{F_{c}^{2} }}{{F_{0} }}} \right)^{\frac{1}{2}} + 1}}$$

Figure 4B shows an illustration of the optimality in quarantine rate given a maximum capacity of quarantine rate $F_{c} = 200$. Both the benefit (B) and cost (C) increases with $F_{\alpha }$ which leads to an optimal $F_{{\alpha^{{}} }}^{opt} \sim 60$ where (B–C) is maximized (Fig. 4B). The optimal value is much below the maximum quarantine capacity of 200. Finally from Eq. (1) the optimal lockdown removal ($\rho_{opt}$) to maintain $R_{0} < 1$ is given by

3 $$\frac{\beta }{{\alpha_{2} }}\frac{{\rho_{opt} }}{{\rho_{max} }} = F_{c} \frac{{\left( {\lambda \frac{{F_{c}^{2} }}{{F_{0} }}} \right)^{\frac{3}{2}} + 1}}{{\left( {\lambda \frac{{F_{c}^{2} }}{{F_{0} }}} \right)^{\frac{1}{2}} + 1}}$$

Hence as the quarantine capacity $F_{c}$ is low, the $\rho_{opt}$ would be low (Fig. 4C) which illustrates: as the quarantine capacity is low, the extent of unlock should remain optimally low. Notably, higher value of unlock $\rho$ is possible by increasing $F_{\alpha }$ beyond $F_{{\alpha^{{}} }}^{opt}$ to maintain $R_{0} < 1$, but the overall benefit would be low given the cost of testing. In order to increase optimal extent of unlock $\rho_{opt}$, the maximum quarantine capacity $F_{c}$ is also required to be increased (Fig. 4C) by augmenting the maximum testing capacity. In addition, the optimal benefit of testing would also depend on the intrinsic transmission rate ($\beta$). For instance, owing to higher value of $\beta$, given a maximum capacity ($F_{c}$, the optimally beneficial unlock extent would be low for the USA (Fig. 4C) compared to Austria or Australia with much lower values of $\beta_{{}}$ who could afford a greater degree of unlocking for same $F_{c}$. Similarly, Eq. (3) also shows that the optimal extent of lockdown ($\rho_{opt}$) would increase if the cost of testing ($\lambda$) increases. One effective way to manipulate the extent of lockdown is by varying the frequency of full unlock in a periodic lockdown-unlock^20^ (time interval of full unlock $T_{1}$ followed by a time interval of full lockdown $T_{2}$) within a time cycle of period $T = T_{1} + T_{2}$ (SI section V and Figure S4 for details).

### Agent-based contact tracing model quantitatively connects testing rate to desired unlock extents

**Figure 5:**
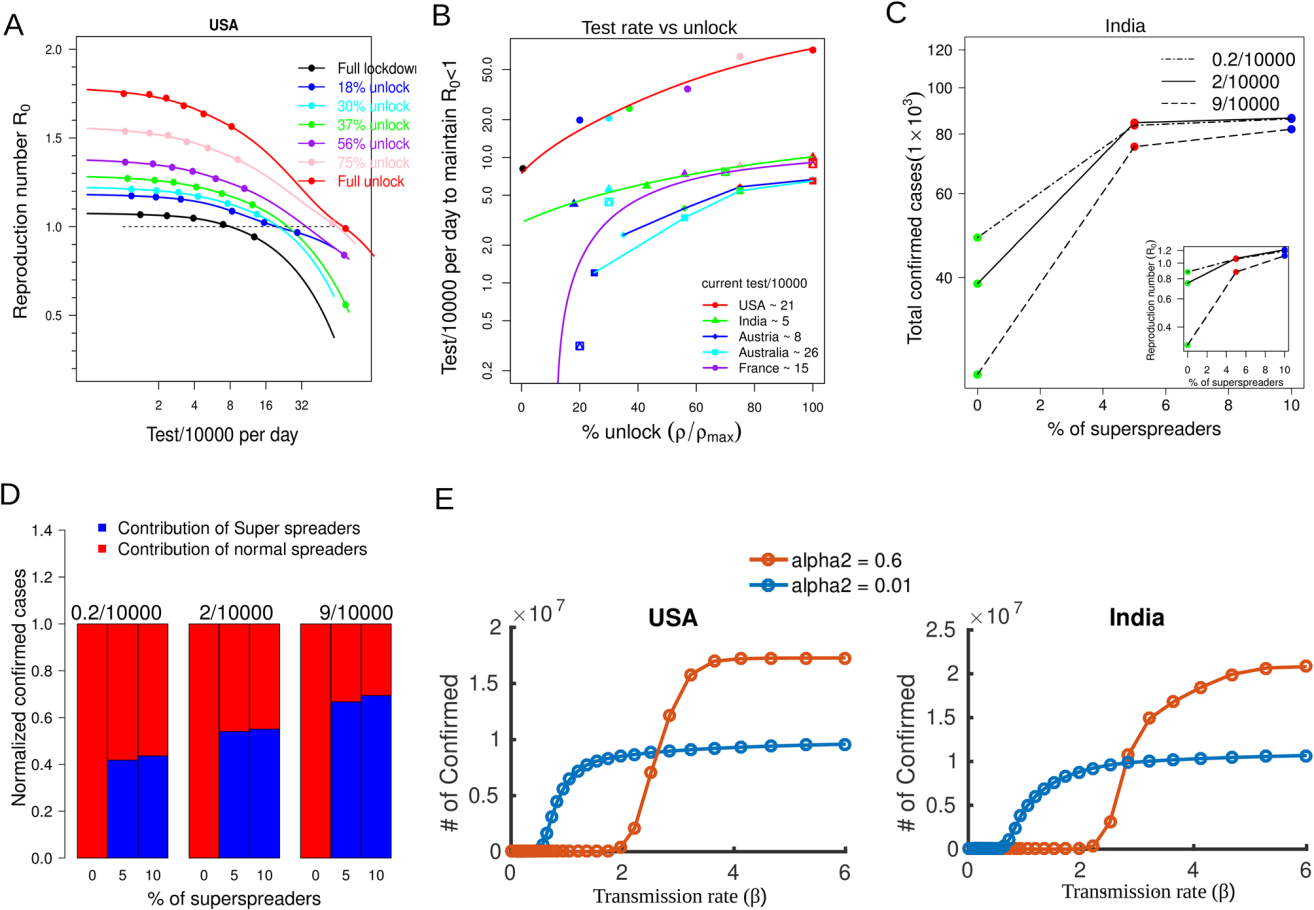
The testing rate and fraction of superspreaders in the population influence the infection rate simulated by the agent based model. (**A**) The $R_{0}$ values based on the agent based model with parameter values of the USA as a function of test/10,000 for different extents of unlock as indicated. The other four countries are shown in supplementary figure S4. The dashed horizontal line exhibits the $R_{0} = 1$ line, intersection of the line with the curves quantifies the test/10,000 required to maintain $R_{0} = 1$. (**B**) The allowed extent of unlock and the corresponding test/10,000 required to keep the $R_{0}$ value less than one calculated from the agent based simulation for the different counties with indicated current test rate. (**C**) The total confirmed cases as a function of percentage of superspreaders simulted for parameter values of India. Different lines indicate simulation conducted at different testing rates as indicated. (The inset shows the corresponding $R_{0}$ values at different percentages of superspreaders). (**D**) The barplot indicates the contribution of superspreaders and normal spreaders over the total number of confirmed cases as a function of the percentage of superspreaders at 3 different testing rates 0.2/10,000, 2/10,000 and 9/10,000, as indicated. (**E**) The relationship between the transmission rate and the total confirmed cases at two different values of quarantine rate as indicated for two representative countries, the USA and India.

The ODE modeling frameworks used to study the dynamics of infection (including ours) cannot model contact tracing explicitly, hence it cannot connect the number of testing required to achieve a certain quarantine rate. To address this gap we have next developed an agent-based model (ABM) to introduce testing through contact tracing, such that a quantitative understanding on the required number of testing per unit population can also be derived (details in methods), in a country specific manner. In this hybrid modeling approach the agent-based stochastic version of the SEIQR model was simulated using country specific infection parameters obtained via calibrating the ODE model to country specific data (methods). In the ABM model infection spreads outward, starting with one infected agent at the center (Figure S5). The selected model for the parameter values of the USA, for instance, shows as the testing rate increases the maximum confirmed cases also increases (Figure S6A, qualitatively in agreement with data shown in Fig. 1C), but this also leads to the containment of infection much earlier causing a reduction in the reproduction number during different partial unlocks (Fig. 5A). The results are comparable for other countries as well (Figure S6B-D). We next used an interpolation method based on cubic spline fitting (smooth.spline from R) to determine the minimum test rate ($N_{test}$) required to keep the $R_{0}$ < 1 during each partial unlock as indicated in Fig. 5A (methods). This calculation results in a monotonic increase in the required testing rate as the extent of unlock gradually progresses from full lockdown towards full unlock (Fig. 5B). The exact values of required testing rates vary among countries for different partial unlocks depending on country specific transmission and quarantine rates (Fig. 5B). It is noticeable that due to low transmission rate before lockdown, countries like Austria, Australia are estimated to be able to unlock by 80% with their current testing rates, whereas for the USA, the current testing rates need to be ramped up at least by 3 times to achieve an unlock of 80%. In fact, this apparently projected connection between the agent-based model and the analytical calculation is justified by the observation that the quarantine rate required to maintain $R_{0}$ at a value of 1 from the analytical calculation follows a linear scaling relation with the corresponding testing rate required to maintain $R_{0}$ at 1 from the agent-based model (SI V for details).

### A small fraction of superspreaders can dramatically increase infection spread during lockdown

The burden on the testing rate capacity can be essentially reduced by keeping the transmission rate low (e.g. Austria) implemented through strict standard measures of social distancing, wearing face masks, etc. However, even during the full lockdown, a small fraction of superspreading individuals^22^ can substantially undermine the diligent efforts exercised by the majority of the population. The superspreaders are defined as having a low quarantine rate and high transmission rate compared to the regular spreaders. A recent detailed contact tracing analysis focusing on a couple of states in India demonstrates that during the full lock down period a small fraction of superspreaders was responsible for transmitting 80% of reported infection, so even with rigorously planned full lockdown $R_{0} < 1$ could not be achieved^22^. Such contributions of superspreaders may generally explain why in most countries full lockdowns could not contain the infection effectively. Using our agent-based contact-tracing model we next simulated different scenarios where a population comprises different proportions of superspreaders.

Our simulations suggest that a small increase in the fraction of superspreaders can drastically increase the number of confirmed cases (Fig. 5C & Figure S6F, simulated with parameter sets specific to India) and also the $R_{0}$ (Fig. 5C-inset) when the testing rate is kept high to best track the spread. Notably, after the superspreader fraction is more than ~ 5% the impact of further increase in supersprader fraction is minimal, suggesting, a sharpe switch-like saturating phenomenon driving the superspreader effect which suggests that it is critical to maintain a minimal number of superspraders during the lockdown, for R0 < 1 (Fig. 5C). For instance, our simulations suggest that a small number of superspreaders (5%) contribute to the spreading of the majority of the infection (~ 70% when the testing rate is 9/10,000, Fig. 5D) and the fraction of total infection for 5% superspreader fraction is marginally less compared to the condition when superspreader composition is 10% in the population (Fig. 5D). The total number of confirmed cases for 5% or 10% superspreaders are also comparable and are distinct from the scenario when the superspreader fraction is 0% (Figure S6F, shown for India). In fact, we observed that the fraction of superspreaders required to have a 50% contribution in total infection over a period of time can be as low as 1% for India, whereas for Austria 3% superspreaders are needed for the same effect (data not shown). Here, Austria owing to a lower intrinsic transmission rate is able to tolerate more superspreaders whereas a very small superspreader fraction in countries like India (or the USA) with high transmission rate can result in a dramatic increase in overall infection. In order to better understand the mechanism for this switch-like behavior, especially at a high quarantine rate, we conducted deterministic simulations at different values of transmission rates ($\beta$). The total confirmed cases increase in an ultrasensitive switch-like manner at a high quarantine rate $\alpha_{2}$ (where $\alpha_{2}$ is the mechanistic equivalent of testing rate in the agent-based model) while the increase is more gradual at low $\alpha_{2}$ (Fig. 5E). This switch can be understood as a consequence of the inherent systems-level positive feedback loop that drives the infection spread in epidemic outbreaks where testing and quarantining are implicit components. Typically the infected and yet not quarantined fraction of the population is the driver of the positive feedback loop (by exposing more subjects to infection) where high quarantine reduces and low-quarantine enhances the strength of this loop. While rigorous testing and quarantining is a necessary measure to contain the infection, it can only delay the onset of the exponential phase of infection spread and movement of superspreaders helps reach the exponential phase faster by fueling the strength of the positive feedback loop to reach the switch threshold. In the ODE model, the superspreaders and regular spreaders would have $\beta$ values above and below the switching threshold (Fig. 5E), respectively, during the lockdown. This will lead to an overall dramatic increase in the number of infected populations owing to the superspreaders' contribution. This analysis thus suggests that in order to leverage the benefit of augmented testing capacity, tracing the movement of superspreaders appears as crucial as the implementation of other standard social distancing measures.

## Discussion

The COVID19 epidemic has disrupted normal life in an unprecedented manner in almost every corner of the world. Similar outbreaks, with different infective capacity, were reported earlier, for instance, the basic reproduction number or $R_{0}$ for COVID19 is comparable to the SARS-cov^26,27^ and much higher than the MERS infection^28,29^. In this study, we quantitatively explored the COVID19 outbreak and its relation with quarantine measures in several countries using both deterministic and agent-based models coupled to analysis of country specific metadata. We first employed a deterministic (ODE based) SEIQR epidemic model and fitted the trajectories from 50 countries at different stages and starting times (day) of infection. We then built an agent-based stochastic model^30^ to implement contact tracing by utilizing the best-fit parameters from the ODE based SEIQR model and implement them in the agent-based model while ensuring a quantitative scaling between both models. The primary goal of utilizing the two distinct modeling approaches is to connect the testing rate to the unlock process since contact tracing can not be implemented in ODE based epidemic models. Earlier studies discuss the limitations of ODE based models and agent-based models^32^, but studies connecting both modeling approaches especially in the context of the COVID19 epidemics to understand the relation between testing rate and unlock measures are not reported in the literature. Here, feeding the country specific epidemic parameters from the ODE model into the agent-based model, we derive country specific optimal testing rates through contact tracing. Such analysis aims to guide different degrees of unlocking to open up economies in different countries since long-term lockdown is impractical and detrimental to the economy of any country^31^.

Typically, the actual infected number of people is expected to be higher than the sampled ones and limitations like that pose challenges for devising accurate mathematical models^32^. The actual susceptible population size is thus mostly unknown and it could often be different from the tested population size, so the number of infected people from the data only captures the tested sample when the total number of tests is less than the total population of a region/country. This is also one of the reasons why increased testing rate is critical in better capturing the magnitude of the infection (and not only the nature of the dynamics, the dynamic features of the infection spread can be robustly captured even with a relatively smaller tested population size) that can lead to devising more accurate epidemic models with better predictive capacities in early stages of the infection. In addition, because of the asymptomatic nature of the infection^33^, the need to quarantine the maximum number of infectious people through testing is equally important to successfully contain the infection spread. Apart from the testing rate, other factors like immunization^34^, age^35^, sex^36^ medical facility and specific virus strains^37^ may play important roles both in transmission and fatality but testing rate is the only variable amongst all these critical factors that can be manipulated immediately post infection via human intervention.

In countries like India where population density and size outmatch the healthcare infrastructure, an early implementation of lockdown was critical in slowing down the spread of the infection. Similarly, in the early days of CDOVID19 spread, delay in lockdown implementation had a catastrophic impact in Italy or Spain despite adequate health infrastructure per capita, highlighting the importance of implementation of early and rapid measures post infection irrespective of healthcare infrastructure. This is further highlighted by countries like India and the USA where unlock measures immediately witnessed a dramatic increase in infection spread.

Reopening the economy was also an impending necessity in all countries under lockdown. Here, utilizing quantitative knowledge on infection and lockdown dynamics from the ODE model and feeding the country-specific best-fit parameters into the agent-based model, we propose strategies for optimal testing and quarantining of the infected subjects to maintain $R_{0} < 1$ during the unlock periods. We quantitatively demonstrate the quarantine and testing that can be optimally adjusted (post lockdown) to minimize the infection spread. We demonstrate that the effective increase in testing mediated quarantine measures has to be country specific, primarily depending on the transmission and quarantine rates of a country. Already, many countries have taken initiatives to accentuate the testing capacities either through developing more testing facilities^14–16^ or through designing efficient pool testing algorithms^38,39^. Our study can additionally help facilitate more informed planning of such testing processes where, for instance, if a goal of 30% unlock of economy is set by a given country (or a province in a country), then the respective increase in testing rates to achieve such unlock goals can be predefined as the minimal testing rate target. In an unprecedented efficiency, multiple vaccines are rolled out now, which will eventually aim to contain the COVID19. But it is still relatively unclear how the rapidly evolving variants of the virus will impact the efficacy of different types of vaccines as the vaccines have distinct mechanisms of target engagement. Further, many developing or underdeveloped countries may not have access to such widespread immunization programs to vaccinate their entire population till at least 2022^40^. Thus, systematic planning of testing and designing of lockdown-unlock measures remain key factors in containing the current spread of COVID19 in specific and perhaps in case of similar such epidemics in the future.

Periodic lockdown^21^ is another efficient way to implement partial lockdown which best works if the $R_{0}$ value during the lockdown is much lower than 1 maintained typically via rigorous testing and quarantining. However, in many developing as well as developed countries, it became almost impossible to limit the mobility of people even during lockdown^11,25^, thus, maintaining a low $R_{0}$ during lockdown remains a challenge. A small unidentified fraction of the exposed population during the unlocks can potentially remain unidentified due to the long incubation period characteristic of COVID19. Indeed, we saw a higher surge of a second wave all over Europe that qualitatively resembles the second wave of Spanish flu pandemic of 1918^41^. A recent landmark study highlights how a small fraction of unidentified superspreaders dramatically accelerated the spread of infection in two states of India even during nationwide lockdown^22^ and undermined India’s advantage of early lockdown. Looking at the trend of infection dynamics across the world, this could be true to multiple other countries as infection numbers kept on growing constantly even during the lockdowns. Thus, the discovery of superspreader mediated infection spread via contact tracing can be a critical component of lockdown implementation itself which further underscores the advantages of our proposed strategy of optimally augmenting the test rate through contact tracing. Additionally, the emergence of a few more new strains may also have accelerated the upsurge of confirmed cases^45^. In this study, using an approach of cost–benefit optimization we derived a data-driven strategy that aims to achieve $R_{0} < 1$ during unlocks which will perhaps also reduce the severity of uncontrolled future outbreaks. A general property of epidemic outbreaks such as COVID19 is that they are fueled by the strength of an implicit positive feedback loop connecting the infected population to the susceptible population. Such positive feedback can promote switch-like responses especially when the testing/ quarantine rates are high. Our simulations suggest a small fraction of superspreaders (with high $\beta$) can exploit the switch-like response capacity of the system to trigger a major increase in infection number during the lockdowns. As the superspreader fraction increases beyond a threshold even for a high testing rate scenario a sudden increase in the number of confirmed cases can arise due to overall change in the strength of the positive feedback loop. Thus, the containment of superspreaders during the lockdown would be critical to maintain a low $R_{0}$ such that subsequent unlocks can be implemented in an optimal manner, especially in densely populated cities where the transmission probability is higher. In fact, the recent upsurge of infection spread in several regions even after the launching of vaccination programs^8^ reiterates the warning that our complacency in obeying all social distancing and quarantine rules out of desperation to return to pre-pandemic life may incur fatal consequences. Finally, if this COVID19 pandemic is successfully contained in the coming months due to improvements in the efficacy of vaccines, the strategy we propose here may still facilitate better containment of future such outbreaks.

One of the limitations of our study or similar such studies used commonly to model infection spread in epidemics like COVID19 can be the assumption that the population is homogeneously distributed over a region/country with an equal number of contacts for every individual. In the real world the contact network in many cases follows a scale-free structure^46^. In fact, a recent study has shown that incorporating such detail indeed improves model prediction accuracy^47^. However, it is also suggested that such a real-world network would slow down the spread of infection^46^. Thus, in our study, the prediction accuracy is expected to be less with a homogeneous contact network and the required testing rate would plausibly be overestimated (but not underestimated) due to this assumption. Different realizations of the contact network can easily be introduced in our ABM framework, but it will also increase the complexity of the current model with inclusion of additional parameters. Future extensions can systematically explore the effects of different types of real-world contact networks and their meaningful combinations on infections spread as well as testing rate required to maintain $R_{0} < 1$. Another model assumption pertains to the fact that all the infected population will eventually be quarantined in the ODE model. This assumption is derived from the observations that during the COVID19 outbreak the capacity for asymptomatic people to spread the infection is much less compared to the symptomatic people^33^ hence the positive feedback loop connecting the infected to the susceptible individuals is primarily driven by the infected and symptomatic individuals, an assumption also commonly used in epidemic models used to quantitatively model COVID19 infection^24^. Here, as the quarantine rate would determine the $R_{0}$ value, a high quarantine rate would prevent the infected population from spreading the infection within a relatively shorter time scale while a low quarantine rate would allow the infected individuals to keep on spreading the infection. Based on the country specific quarantine rates the infected (and symptomatic) individuals will be quarantined at different rates, but in the duration between being infected and before being quarantined the individuals will continue to infect others, country specific dynamics of which is quantitatively captured by our country specific models. However, in a scenario where the asymptomatic subject driven infection spread is significantly higher, the models built with the current assumptions (including ours) will not capture the true dynamics of infection spread and would require extension to explicitly incorporate the asymptomatic compartment. In such scenarios understanding the true dynamics of infection would require excessive testing through contact tracing^48^.

For the ABM model, since we are considering testing through contact tracing, if we start with a very high testing rate, the infection rate would be low which would require a small number of infected agents to be traced and tested. On the other hand, if we start with a very low testing rate, many exposed/infected individuals will remain untested. Hence in both low and high testing rate scenarios the total population will not be tested. In addition, since we also have a quarantine rate of infected population without any contact tracing and testing, similar to the ODE model, all the infected individuals will be quarantined eventually albeit slowly in the ABM model as well.

## Materials and methods

### Calculation of doubling rate

The doubling rate is calculated at each time point over the trajectory by taking the daily cases at that time and the next day. For an exponential growth of the infection, $N\left( t \right) = N\left( {t + 1} \right)exp\left( {r_{d} } \right)$ where N(t) depicts the number of infected people at time t

$$r_{d} = log\left( {\frac{{N\left( {t + 1} \right)}}{N\left( t \right)}} \right)$$

Here t = 1 day. The values in the heatmap in Fig. 1B are displayed in an exponential scale of doubling rate to avoid negative values. So a doubling rate of 1.4 in exponential scale means the doubling time of $\frac{1}{{log\left( {1.4} \right)}} = 3$ meaning it takes around 3 days to double the number of infected people.

### Correlation analysis

For the correlation and significance of the correlation, we used cor.test() function in R. The function uses t-distribution statistics to calculate the p-value of the correlation. The t value of a pair of random numbers with correlation r and number of points n is give by,

$$t = r\frac{{\sqrt {n - 2} }}{{\sqrt {1 - r^{2} } }}$$

The t-distribution provides the probability of t values for the null hypothesis that the mean correlation of the two random variables is zero. If the calculated correlation value falls on the tail of the t-distribution, the correlation is significant. The exact p-value is calculated from the t-distribution table of n degrees of freedom. The calculations are all done by the cor.test() package in R.

#### The SEIQR model

The model comprises of susceptible (S), Exposed (E), Infected (I), Quarantined (Q), Removed (R which contains two compartments ‘recovered’ and ‘dead’).

The equations are

$$\begin{aligned} & \frac{dS\left( t \right)}{{dt}} = - \beta *\rho \left( t \right)*\omega \left( t \right)*S\left( t \right)*I\left( t \right) \\ & \frac{dE\left( t \right)}{{dt}} = \beta *\rho \left( t \right)\omega \left( t \right)S\left( t \right)I\left( t \right) - \alpha_{1} E\left( t \right) \\ & \frac{dI\left( t \right)}{{dt}} = \alpha_{1} E\left( t \right) - \alpha_{2} I\left( t \right) \\ & \frac{dQ\left( t \right)}{{dt}} = \alpha_{2} I\left( t \right) - \gamma_{d} Q\left( t \right) - \gamma_{r} Q\left( t \right) \\ & \frac{dR\left( t \right)}{{dt}} = \gamma_{r} Q\left( t \right) \\ & \frac{dD\left( t \right)}{{dt}} = \gamma_{d} Q\left( t \right) \\ \end{aligned}$$

where S(t), E(t), I(t), Q(t), R(t) and D(t) are the susceptible, exposed, infected, quarantine, recovered and dead population at time t respectively. ρ(t) is the lockdown function and $\omega$(t) is the time when infection starts in a given country after the first day of detection of the infection date as reported in WHO website (hence simulation start time corresponds to 22nd Jan, 2020 based on the WHO report).

The lockdown function ρ(t) is given as

$$\rho \left( t \right) = \frac{1}{{1 + \left[ {\frac{K}{{1 + exp\left( {tc - t} \right)}}} \right]^{S} }}$$

Here K = Effect_lockdown_Country which determines the maximal effective lockdown for a given country, for instance K = 1, 2 and 3 would result in 50%, 66.6% and 75% maximal lockdown, respectively, during the lockdown. Here tc = time of lockdown start and S = strength of lockdown that determines the steepness of the lockdown implementation time from no lockdown to the maximal lockdown.

ρ(t) varies between 1( no lockdown) and 0 (full lockdown). $\omega$(t) ensures that the model for a specific country is switched on when the infection begins in that country, hence if the detected case in a country is 40 days after Jan 22nd, the model for that country is switched on 35 days post Jan 22nd (assuming mean incubation time of 5 days), during the calibration.

The lockdown is opened by modifying the ρ(t) function such that ρ(t) returns to 1 from its lockdown status to no-lockdown (1) status in a designated time .

### Model calibration

Model calibration involves minimizing an objective function that gives best fit parameter sets for confirmed, recovered and dead populations for a given country, simultaneously. We fitted the time series provided by JHU CSSE at github^43^ to the SEIQR model developed in the study and minimized the objective function using the nonlinear least square fitting algorithm (lsqnonlin) from MATLAB.

It returns x = lsqnonlin(fun, × 0, lb, ub), where x is a vector/matrix of variables whose values are to be determined, for instance, in our case x comprises the country specific infection parameters to be estimated via the fitting the model to the observed infection dynamics for a given country of interest. The function ‘fun’ contains the residuals or sum of squares of the difference between model value and data point for a given time point; the residuals are evaluated for each time point for which data point is available. × 0 is the initial parameter vector that comprises the initial guesses of the model variables and ub and lb respectively represent upper bound and lower bound vectors corresponding to each value in × 0. For instance, to calibrate the confirmed population trajectory for the USA the fitting algorithm minimizes the sum of residuals for [Quarantined_USA + Recovered_USA + Dead_USA] between both model and data. This exercise was carried out for all the 50 countries individually to obtain the country specific infection parameters (a more detailed description is provided in SI II).

### Analysis of the virus genome sequences for clade distribution

We first collected the individual sequenced genome from individual countries and their corresponding clades as reported^43^. Each genome in a particular country belongs to one of the seven clades (G, GH, GR, L, S, V, O) as described^43^. From the data, we determine the frequency of each clade in individual countries by finding the number of sequences belonging to that clade divided by the total number of sequences. To calculate the correlation, out of the analysed 50 countries, we only considered countries having at least 10 sequences in the database.

### Adjusted $R_{0}$ values

The $R_{0}$ values obtained following the ODE SEIQR model fitting are adjusted with respect to the significant parameters from the correlation analysis described in Figs. 3C, D and S3A,B. The adjustment factor is characterised as

$$A = \frac{1}{populationsize}*median\left( {\frac{age*doctors}{{10000}}*\frac{nurses}{{10000}}*\frac{1}{frequencyofGRclade}} \right)$$

The factors which are positively correlated with $R_{0}$ (population size, frequency of GR clade) are placed in the denominator and factors having negative correlation values are placed in the numerator. The adjusted $R_{0}$ values are quantified as

$$R_{0}^{adjusted} = \frac{{R_{0} }}{{A_{n} }}\;{\text{where}}\;A_{n} = \frac{A}{max\left( A \right)}$$

### Agent-based stochastic SEIQR model

We utilized the model structure and calibrated parameters in the deterministic SEIQR model, as described in the previous section to build an agent-based stochastic version of the same^30^. In this model, (the flowchart below) the individual agents are assumed to be located on a two-dimensional lattice of dimension $300 \times 300$. The points on the lattice represent susceptible agents in the population and each individual on the lattice is surrounded by four nearest neighbors at a minimum distance of one. Here, we considered the agents within a radius of seven as the contacts of every agent on the lattice. Thus, every individual agent would be in contact with a maximum of 28 neighbors. The initial patient at zero time is located exactly at the center having a location of (150,150) on this two-dimensional lattice. The infection spreads through persistent close contact with the nearest neighbors. At every time step, all the exposed and the infected agents are selected and 28 neighbors of the individual get exposed to the infection with a probability $\beta$. The value $\beta_{ODE}$ in the ODE model is defined as the number of individuals a particular person infects per unit time. In the stochastic model each individual would infect $28 \times \beta$ people on an average per unit time. Thus, the conversion between transmission rates of the ODE and stochastic model is given by $\beta = \frac{{\beta_{ODE} }}{28}\frac{\rho }{{\rho_{max} }}$. Here we assumed that both the asymptomatic/exposed and symptomatic patients are capable of spreading the infection. The exposed patient in turn exhibits symptoms with a probability $\alpha_{1}$ and the symptomatic infected patient would be further quarantined with a probability $\alpha_{2}$. The quarantined patient finally either recovers or dies with a rate $\gamma$.

The susceptible, exposed, infected, quarantined and recovered agents on the lattice are assigned values 0, 1, 2, 3 and 4 respectively which allow us to track the dynamics of each component separately (Detail flowchart of the algorithm is provided below). To simulate country specific infection dynamics the parameters values for the ABM model were taken as the respective fitted values from the ODE based SEIQR model for a particular country. In order to introduce diagnostic testing, we identified all the symptomatic as well as the quarantined individuals from the pool at a particular time point and selected a fraction ($F_{test}$) of the identified patients for contact tracing. The contacts of each selected individual were traced and diagnostic tests were conducted on them. The close contacts showing positive results were finally quarantined. This procedure is repeated for all the selected individuals. The status of the whole population is updated and the next time step is continued. The simulation was performed for 200 time points which corresponds to 200 days in the real time as the fitted model parameters used in the ABM model (from the ODE model) have units in 1/day. In order to increase the number of testing, the value of $F_{test}$ was increased. The parameter $\rho$ is varied to change the extent of unlocking. However, we assumed that the testing procedure only commences after at least 100 individuals are already infected in the population, in order to introduce an initial delay in responding to the situation. For every infected person, we also keep track of the number of individuals who are getting infected from that particular infected person until that person is quarantined. The number of people infected during the simulation time defines the reproduction number for that individual. The $R_{0}$ value for the whole population is quantified by taking the average over reproduction numbers for all the agents (persons) in the population. The number of tests ($N_{test}$) is also counted at each time point. For a particular values of $\rho$, the $N_{test}$ and corresponding $R_{0}$ value are recorded which enables us to calculate the minimum $N_{test}$ value required to keep $R_{0} < 1.$ The scaling relation connecting the minimum $N_{test}$ and quarantine rate ($\alpha_{2}$) obtained from the ODE model (Eq. (1)) is discussed in SI-VI in detail. A flowchart describing our workflow that also connects the ODE model and the ABM model is given below.
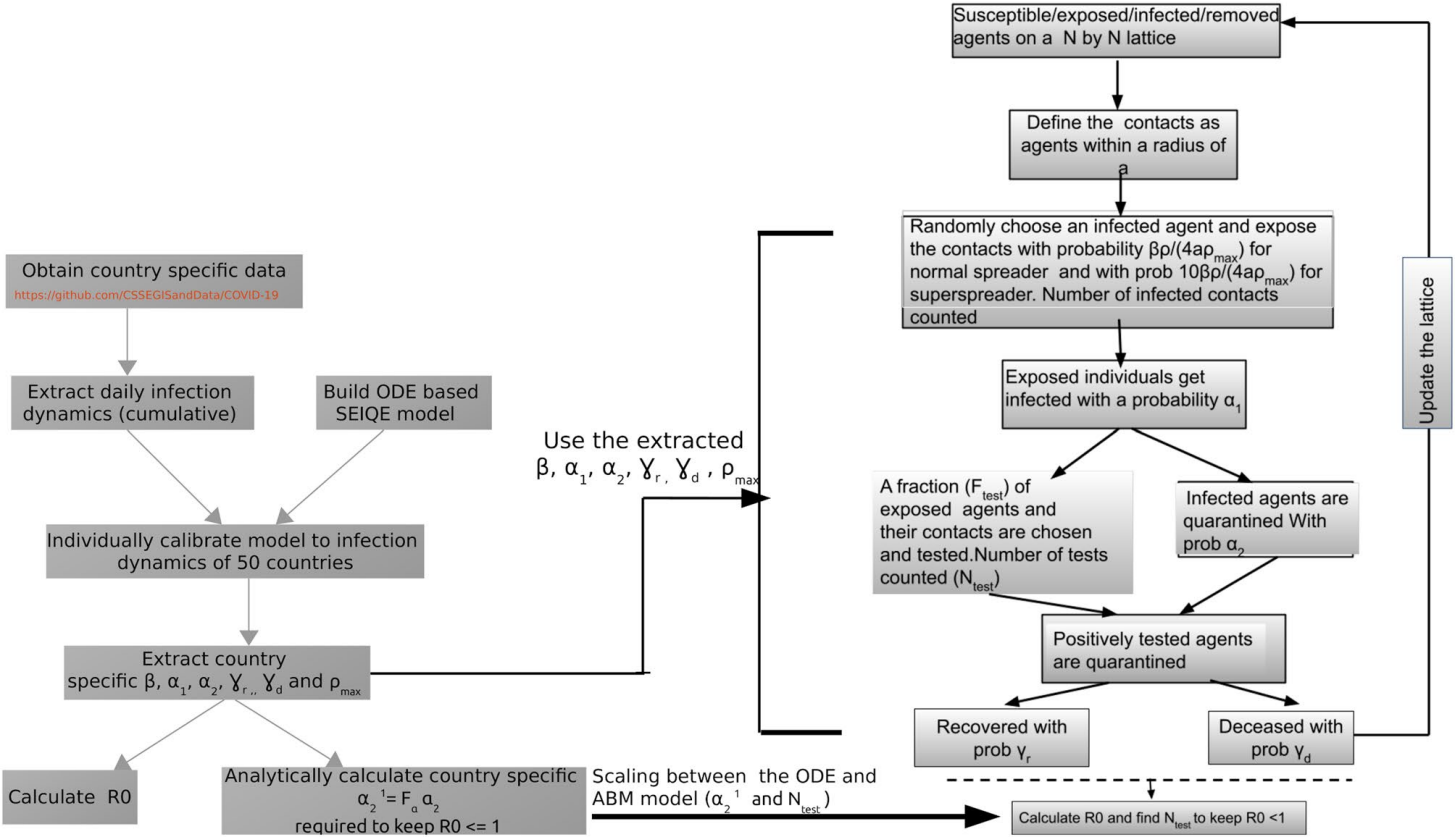


## Supplementary Information

Supplementary Information 1.

Supplementary Information 2.

Supplementary Information 3.

Supplementary Information 4.

Supplementary Information 5.

Supplementary Information 6.

Supplementary Information 7.

Supplementary Information 8.

Supplementary Information 9.

Supplementary Information 10.

Supplementary Information 11.

Supplementary Information 12.

## Data Availability

The data analysis and simulation were performed in R software [R version 3.4.4 (2018-03-15)]. https://cran.r-project.org/bin/windows/base/old/3.4.4/.
