# Supplementary figures and images for: Country-specific optimization strategy for testing through contact tracing can help maintain a low reproduction number (R0) during unlock
Source: Sci Rep. 2022 Jan 7;12:212. doi: 10.1038/s41598-021-03846-z (PMC8742011; doi:10.1038/s41598-021-03846-z)

B

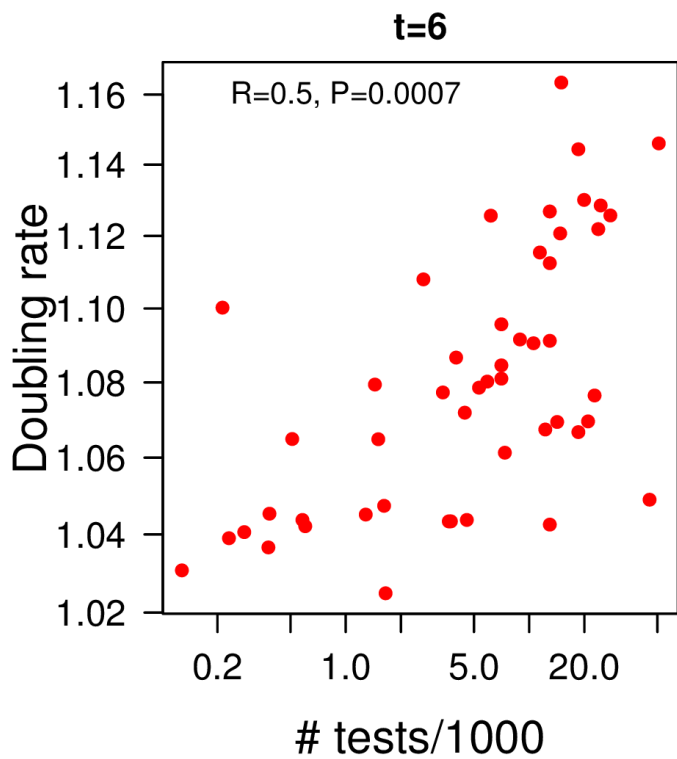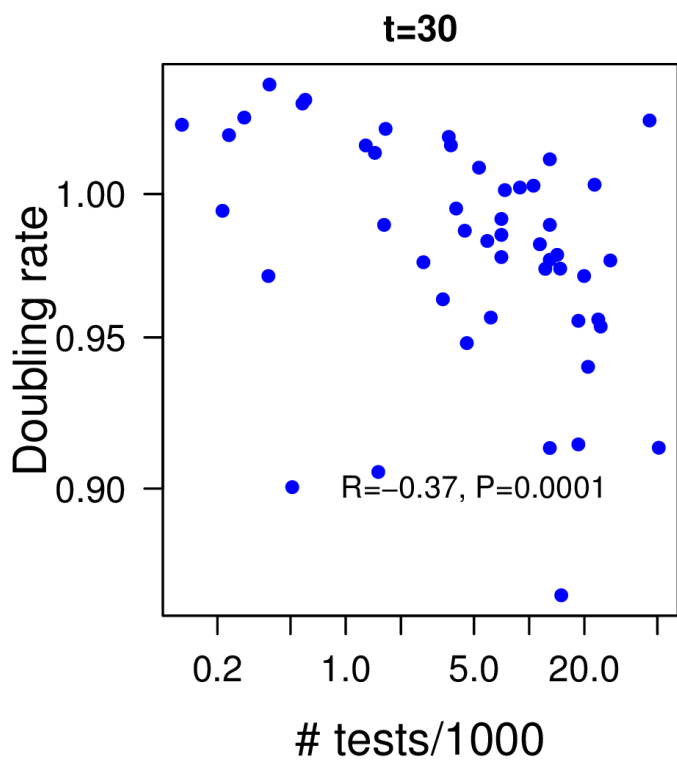

C

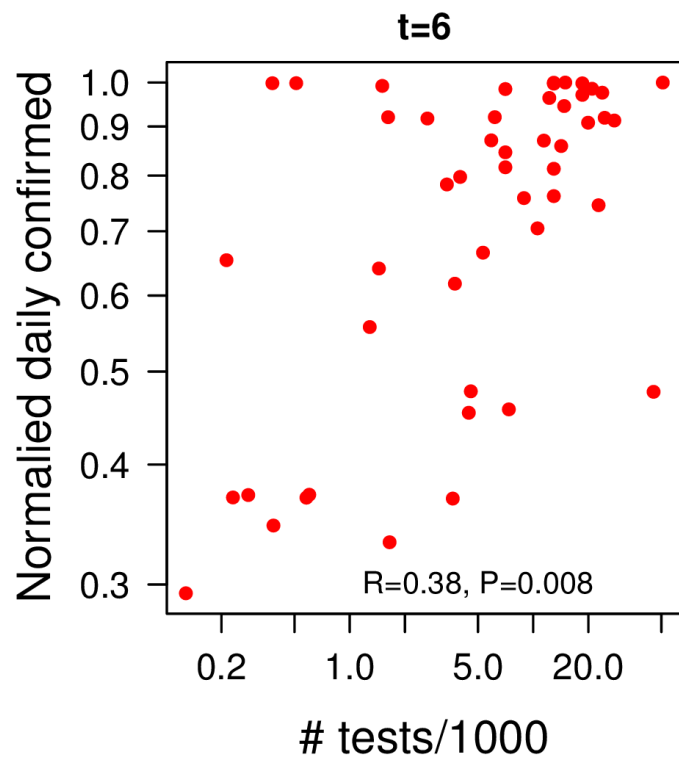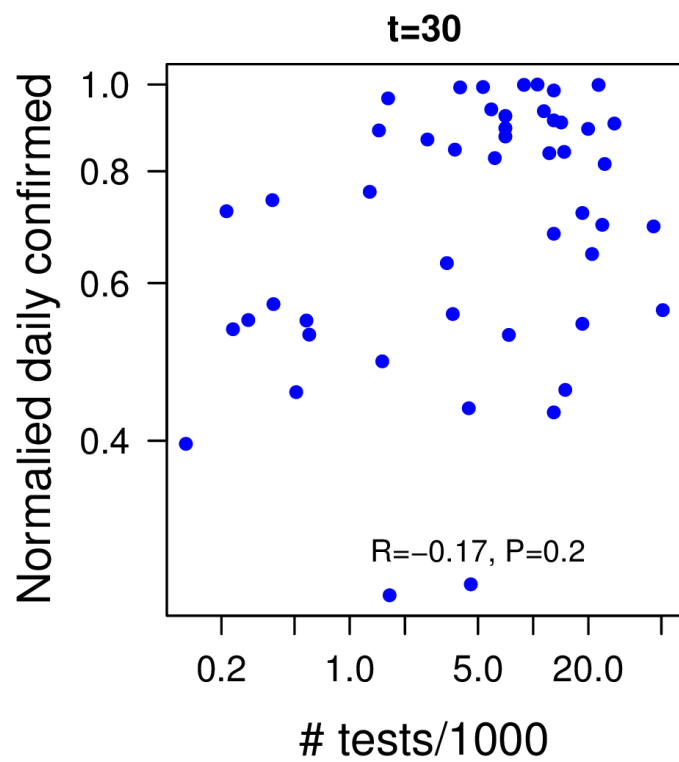

Supplement: Supplementary file 1 — Supplementary Information 1. [file 41598_2021_3846_MOESM1_ESM.pdf]

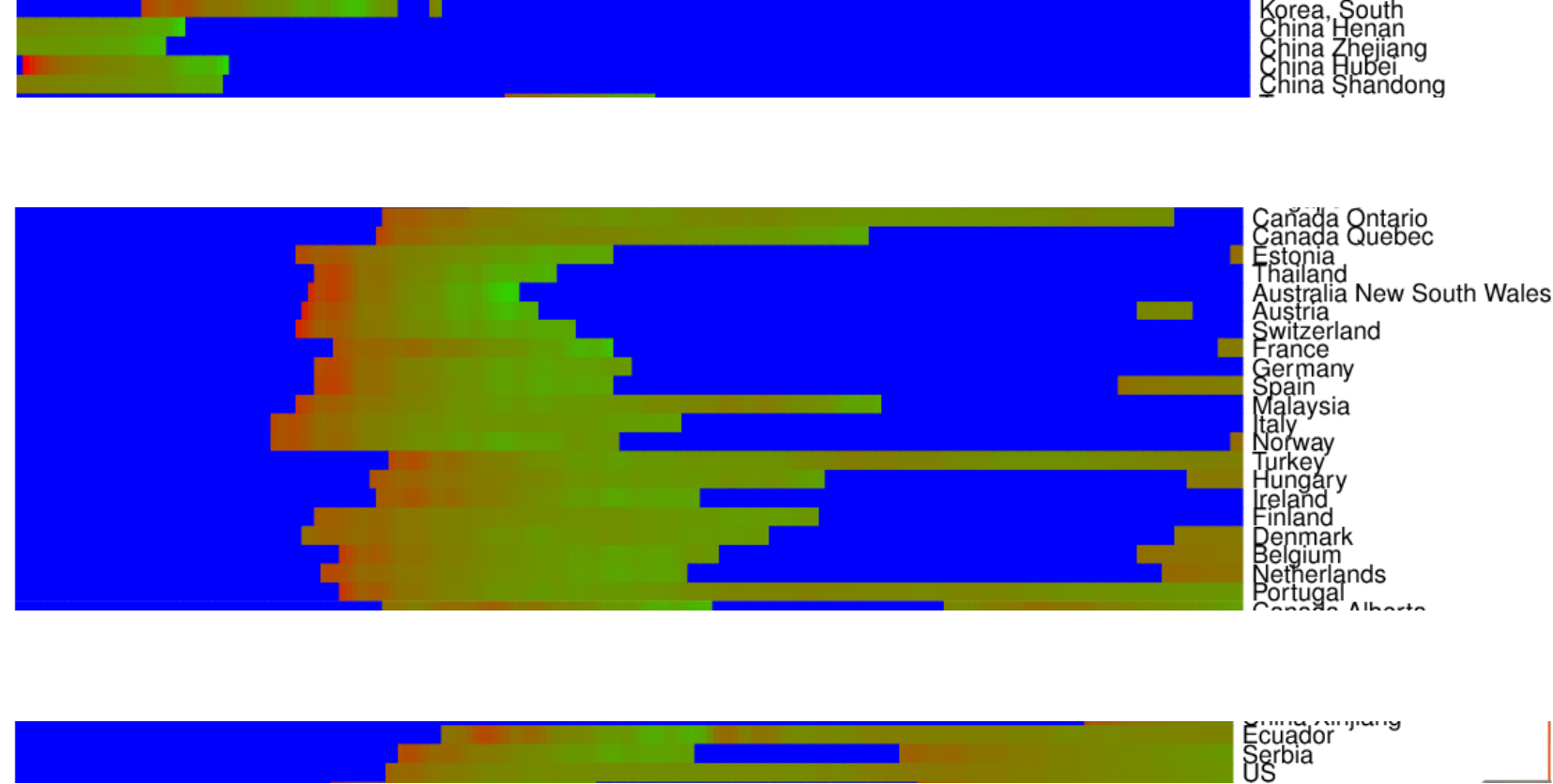

Supplement: Supplementary file 2 — Supplementary Information 2. [file 41598_2021_3846_MOESM2_ESM.png]

Aligned confirmed daily cases

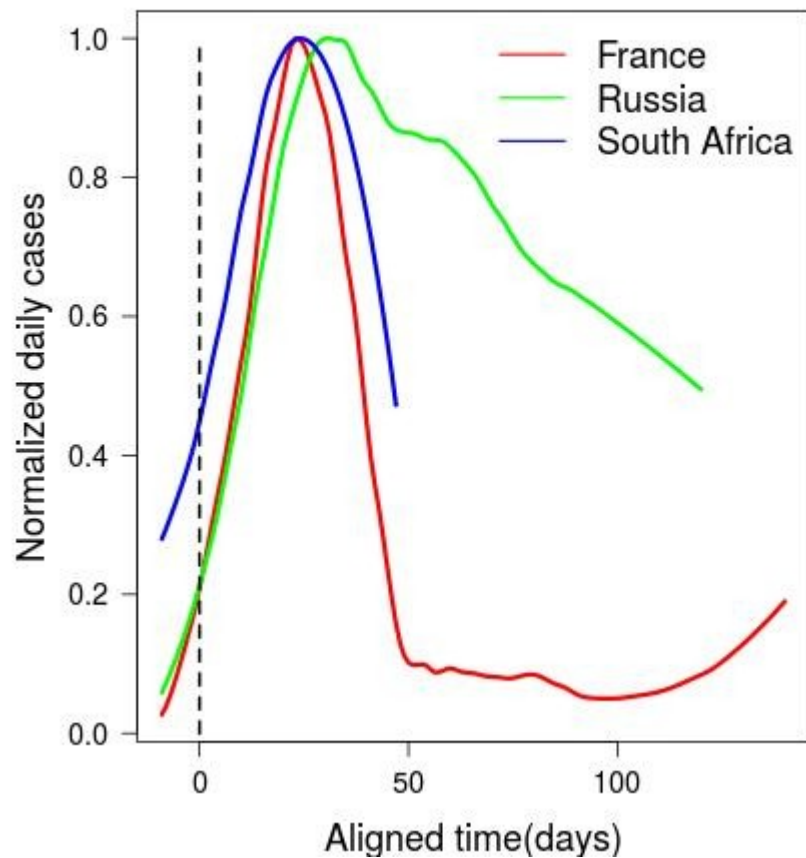

Confirmed daily cases

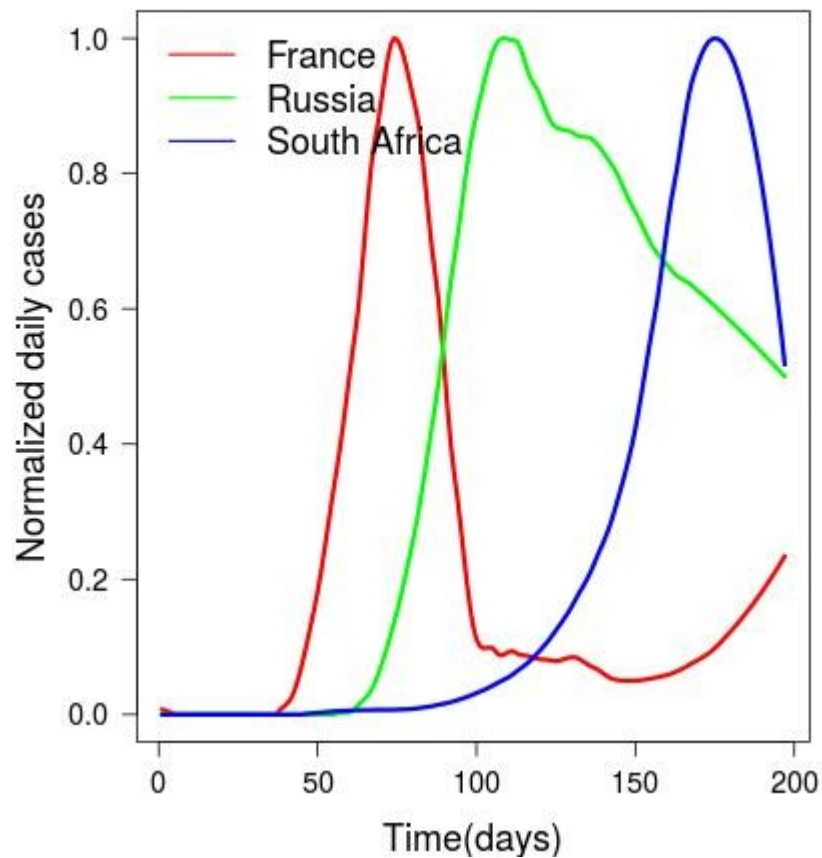

Supplement: Supplementary file 3 — Supplementary Information 3. [file 41598_2021_3846_MOESM3_ESM.pdf]

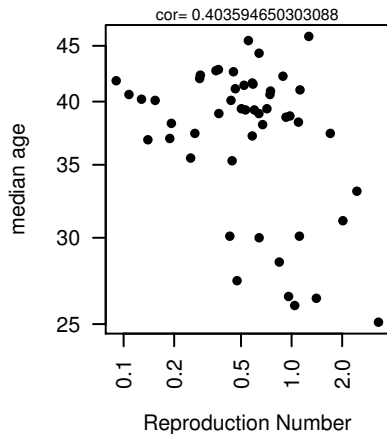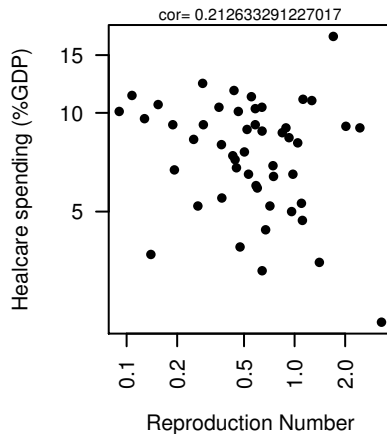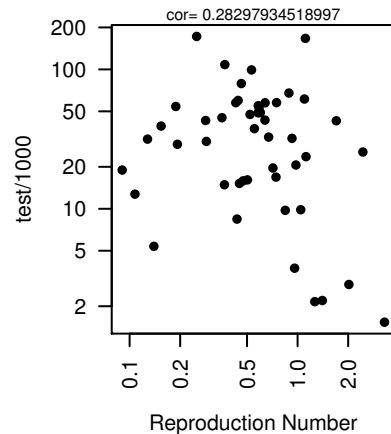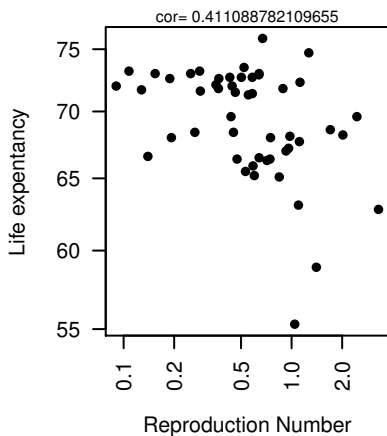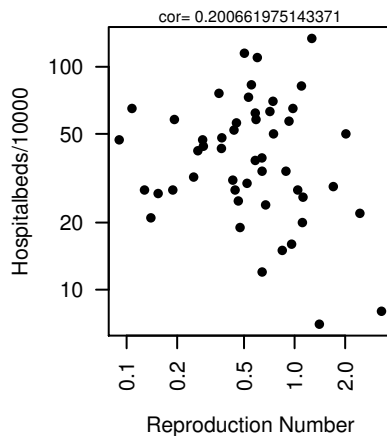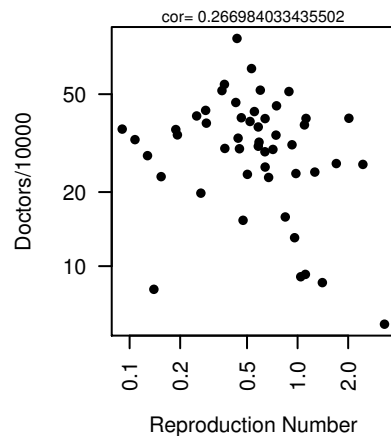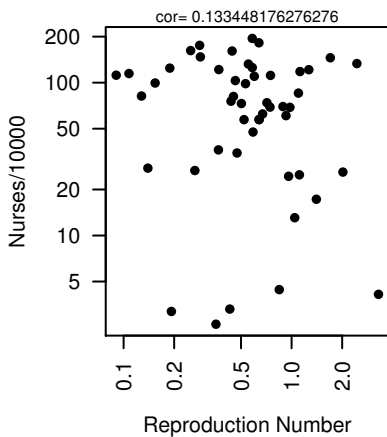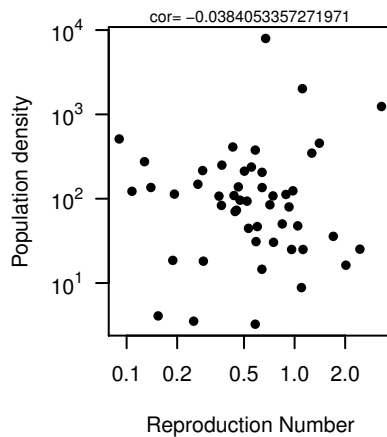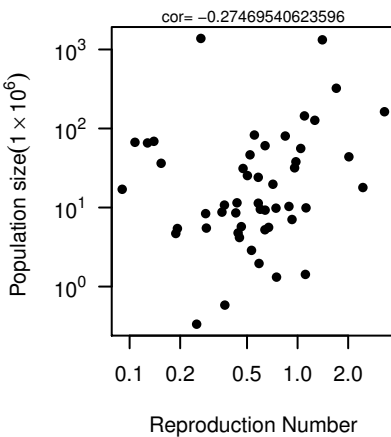

Supplement: Supplementary file 5 — Supplementary Information 5. [file 41598_2021_3846_MOESM5_ESM.pdf]

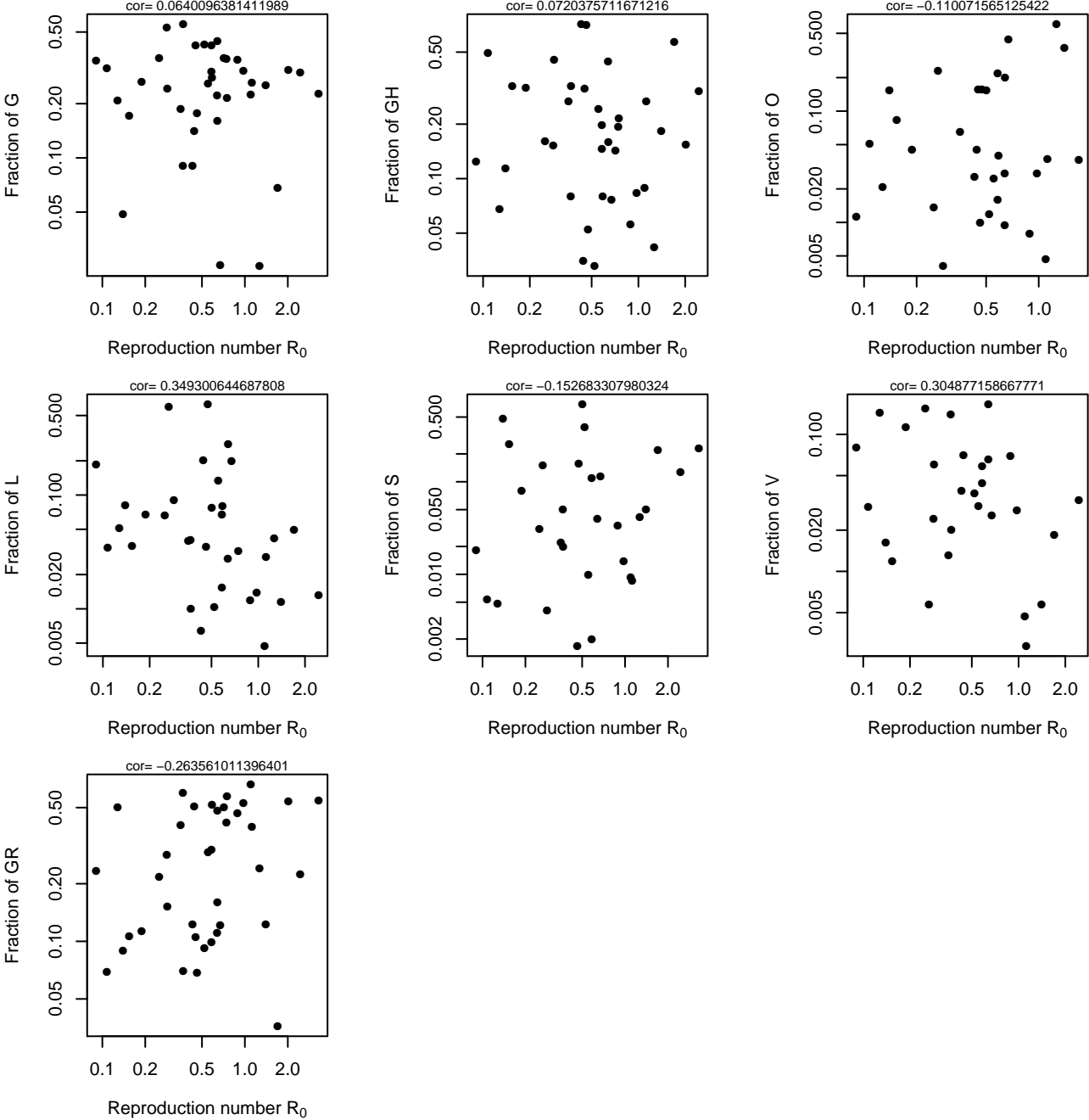

Supplement: Supplementary file 6 — Supplementary Information 6. [file 41598_2021_3846_MOESM6_ESM.pdf]

A

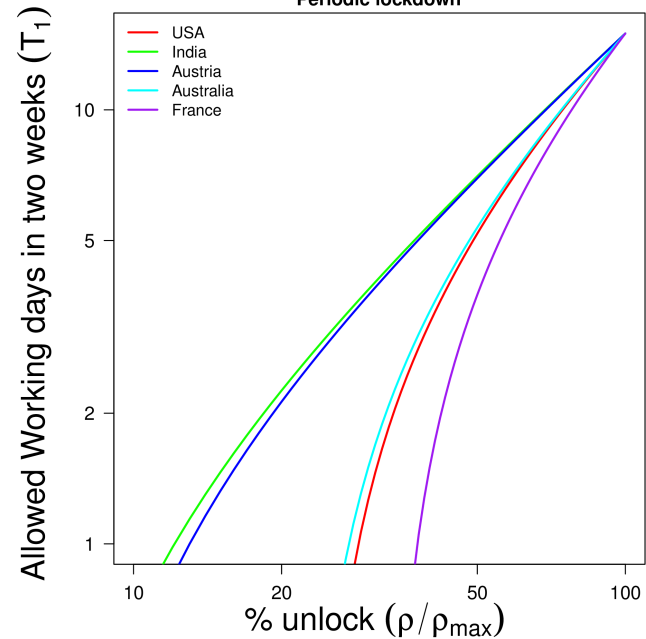

B

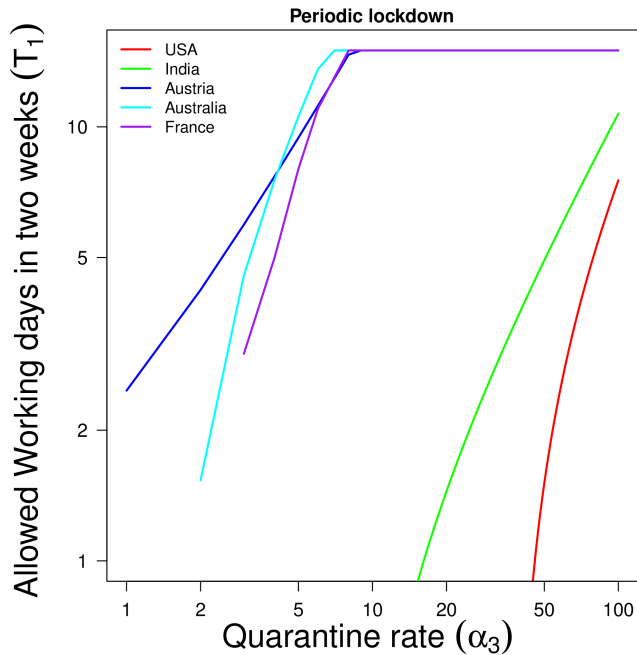

Supplement: Supplementary file 7 — Supplementary Information 7. [file 41598_2021_3846_MOESM7_ESM.pdf]

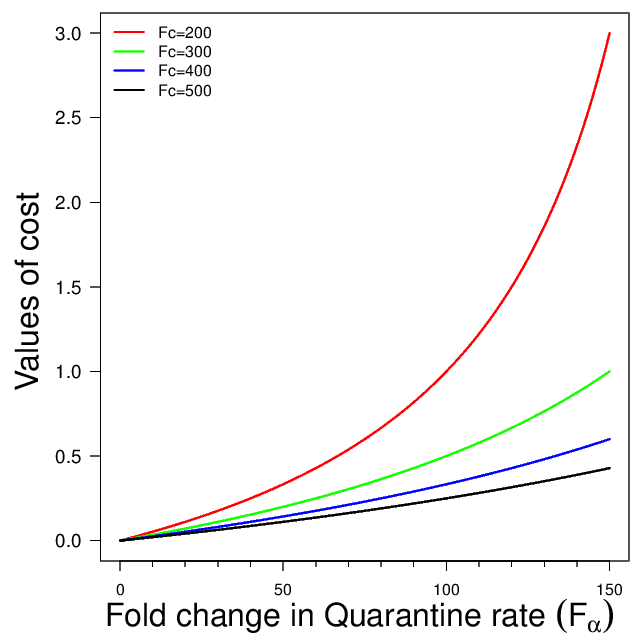

Supplement: Supplementary file 8 — Supplementary Information 8. [file 41598_2021_3846_MOESM8_ESM.png]

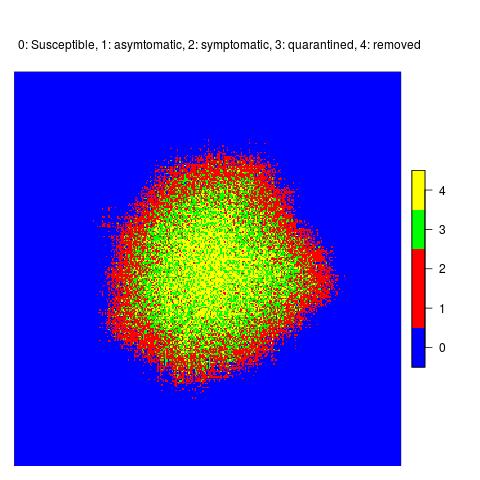

Supplement: Supplementary file 9 — Supplementary Information 9. [file 41598_2021_3846_MOESM9_ESM.jpg]

A

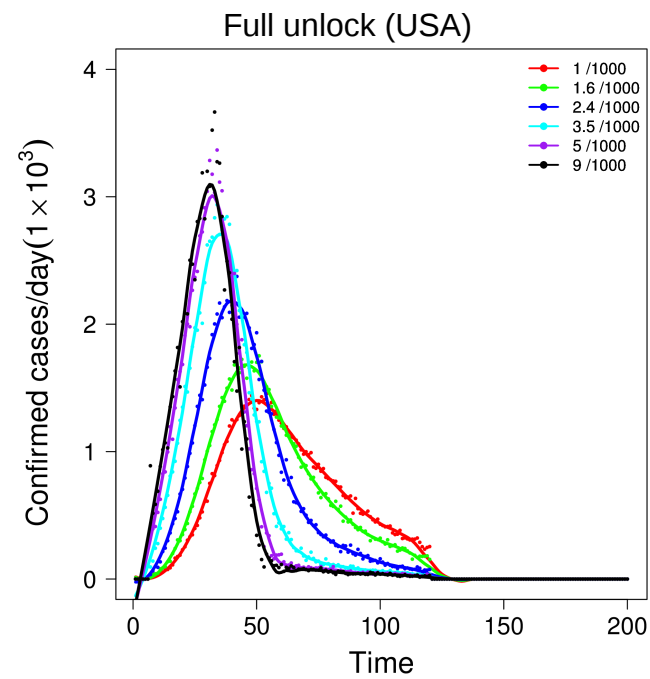

B

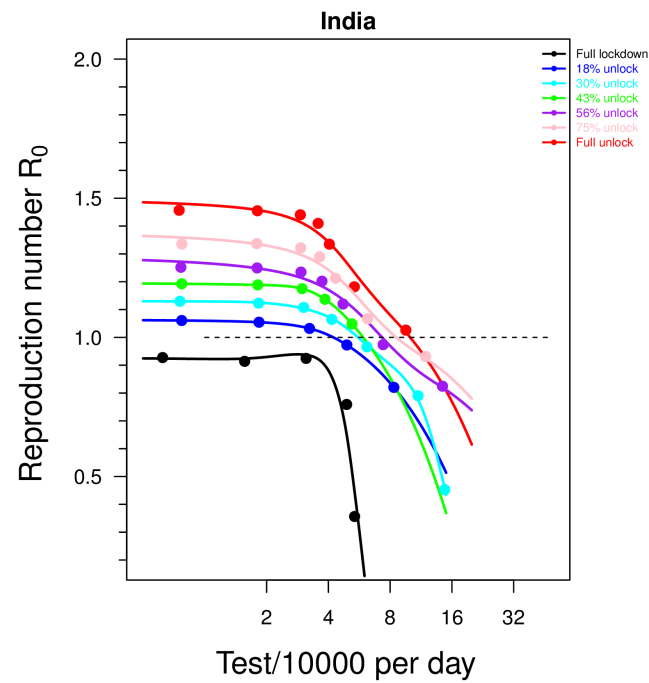

C

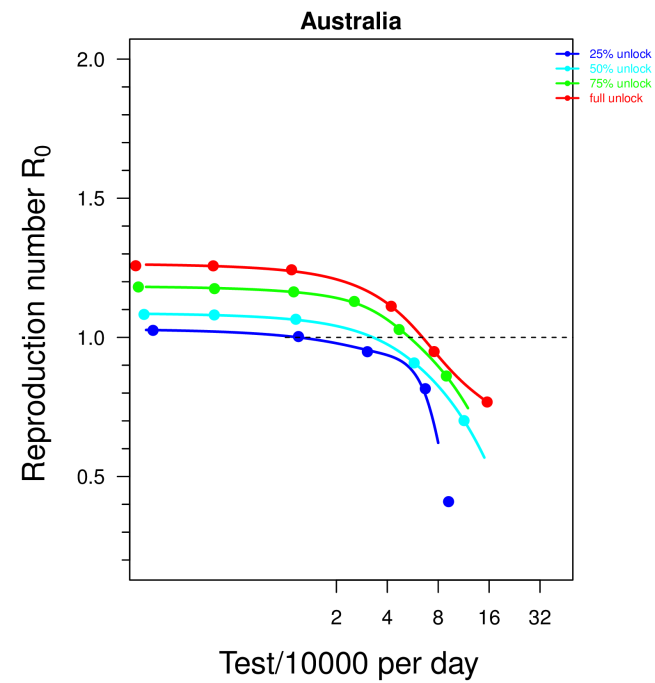

D

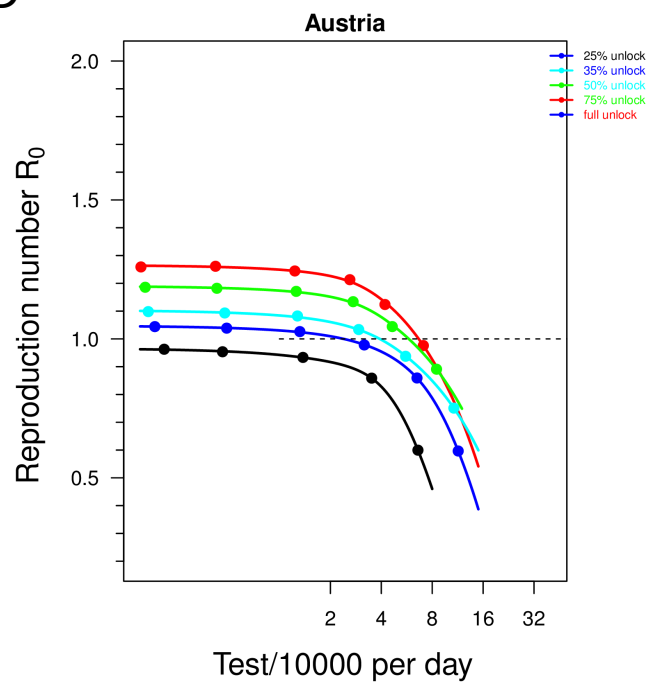

E

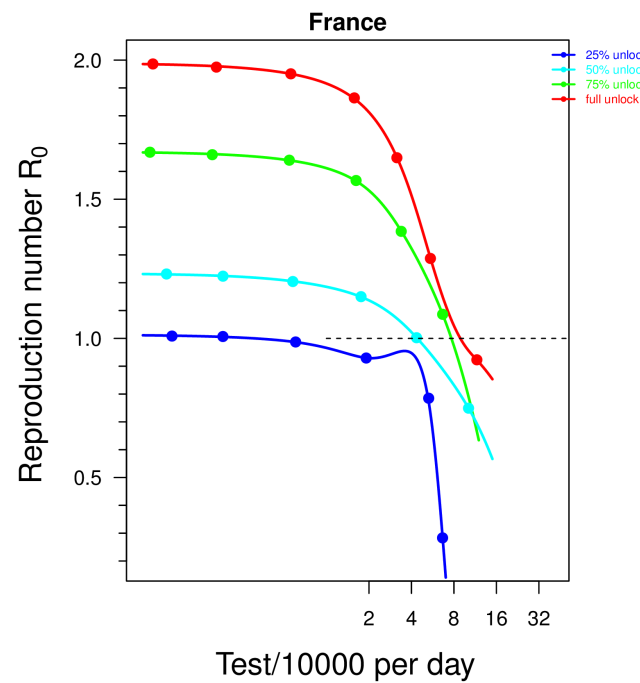

F

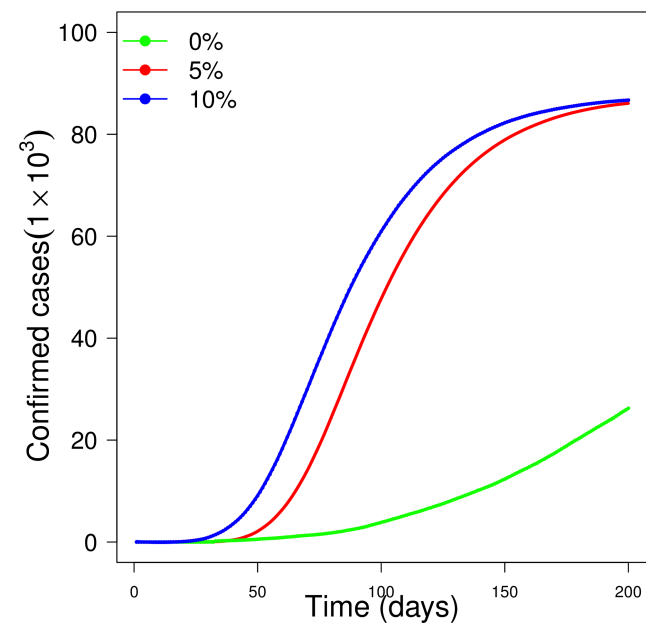

Supplement: Supplementary file 10 — Supplementary Information 10. [file 41598_2021_3846_MOESM10_ESM.pdf]

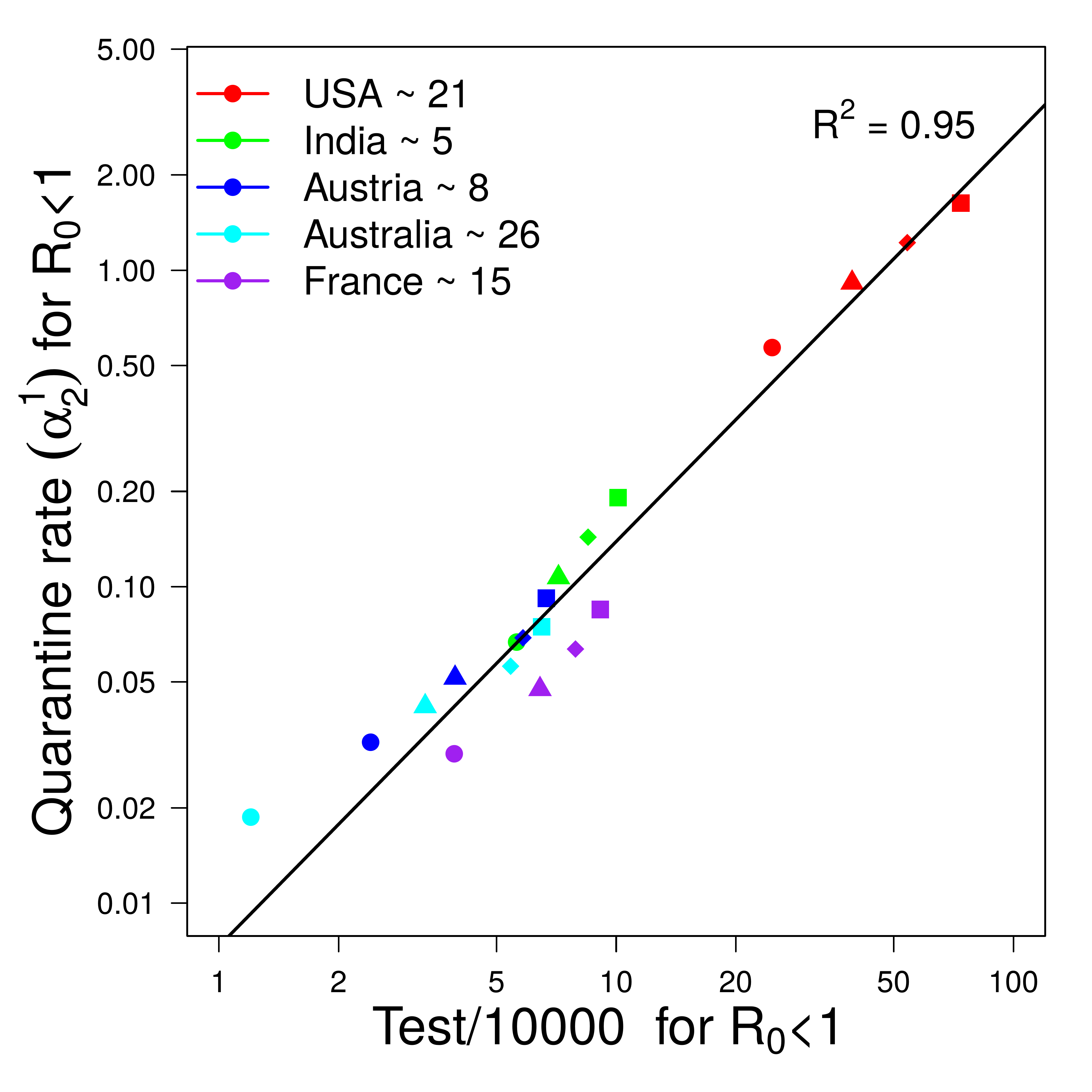

Supplement: Supplementary file 11 — Supplementary Information 11. [file 41598_2021_3846_MOESM11_ESM.png]
