# Supplementary material for: Country-specific optimization strategy for testing through contact tracing can help maintain a low reproduction number (R0) during unlock
Source: Sci Rep. 2022 Jan 7;12:212. doi: 10.1038/s41598-021-03846-z (PMC8742011; doi:10.1038/s41598-021-03846-z)
Supplement: Supplementary file 12 — Supplementary Information 12. [file 41598_2021_3846_MOESM12_ESM.pdf]

Supplementary Information

Country-specific optimization strategy for testing through contact tracing can help maintain a low reproduction number (R_0) during unlock

Uddipan Sarma[1,#], Bhaswar Ghosh[2,#]

[1]Vantage Research, Sivasamy St, CIT Colony, Mylapore, Chennai, Tamil Nadu 600004. India.

[2]Center for Computational Natural Sciences, International Institute of Information Technology, Hyderabad 500032, India

# Joint Correspondence :

Uddipan Sarma : uddipans@gmail.com

Bhaswar Ghosh : bhaswar.ghosh@iiit.ac.in

Content

I. **Alignment of the time courses**
II. **ODE model fitting**
III. **Calculation of of R_0 from the ODE based model**
IV. **Model for optimizing the quarantine rate- the cost benefit approach**
V. **A case for the periodic lockdown**
VI. **Scaling relation between agent based model and analytical model**
VII. **References**
VIII. **Supplementary figure legends**

I. Alignment of the time courses

From the calculation of the doubling rate time courses (Figure 1B), we identified the maximum doubling rate time point for each country and superimposed the time points with each other to align them. To demonstrate this, we took three countries and showed how they are aligned based on the doubling rate with time; in the figure below (Figure S1D), the normalized daily cases are shown and then these three time traces are aligned at the maximum doubling rate. The dashed vertical line at zero in the doubling rate aligned figure corresponds to the maximum doubling rate time point for each country.

II. ODE model fitting

Optimization: The model simulations were calibrated to the observations by fitting values of infection parameters in the model to obtain the trajectories of Confirmed, Recovered and Dead fraction in the population, such that the best possible match between the model and data dynamics can be obtained. To do so an objective function is fitted which minimizes the sum of residuals calculated between model observable and data trajectories. The residual vector comprises the sum of squared difference between model and data for each available data point.

Observable : We have used MATLAB and D2D[2] for model fitting. Model fitting via D2D as explained in the methods section utilizes nonlinear least square fitting algorithm (lsqnonlin) from MATLAB. It returns z = lsqnonlin(fun, z0, lb,ub), where z is a vector/matrix of variables whose values are to be determined whereas lb and ub corresponds to vectors containing the respective lower and upper bounds of the variables subjected to optimization. The fun is the function whose value is to be minimized during the optimization which in this case is the sum of squares between model and data. The observables that are subjected to optimization are as follows

$\text{Confirmed\_population}_{\text{Country\_X}} = \text{simulation\_start}_{\text{Country\_X}} * [\text{offset}_{\text{Country\_X}} + \text{Population\_estimate}_{\text{Country\_X}} * [\text{Quarantined}_{\text{Country\_X}} + \text{Recovered}_{\text{Country\_X}} + \text{Dead}_{\text{Country\_X}}]]$

$\text{Recovered\_population}_{\text{Country\_X}} = \text{simulation\_start}_{\text{Country\_X}} * [\text{offset}_{\text{Country\_X}} + \text{Population\_estimate}_{\text{Country\_X}} *$

$[Recovered_{Country\_X}]]$

$Dead\_population_{Country\_X}$ = $simulation\_start_{Country\_X}$* $[offset_{Country\_X}$ + $Population\_estimate_{Country\_X}$ * $[Dead_{Country\_X}]]$

Where Country_X is a given country whose data is subjected to fitting. The $offset_{Country\_X}$ parameter is ensures that the model trajectory starts with the first reported value of infections for that country at time t = 0 days, and not from zero. For instance, the day 1 in data which is time t = 0 in simulation, is standardized to the first confirmed case in the USA as documented in https://github.com/CSSEGISandData/COVID-19. So the $offset_{USA}$ will be estimated ~ 1 but China had 548 confirmed cases on day 1 which is captured by $offset_{China}$. The component $Population\_estimate_{Country\_X}$ estimates the size of susceptible population in a country during the fitting where the initial guess of susceptible population is provided based on the number of people subjected to testing (extracted from Worldometer : https://www.worldometers.info/coronavirus/). When the number of tests is more than or equal to the population of a country the total population of that country is used as the initial guess as well as the upper bound. This enables us to capture the infection trajectories in countries with order of magnitude difference in population like Austria or India with comparable fit qualities.

The parameter $simulation\_start_{Country\_X}$ is used to ensure the start of model fitting only from the time when the first infection is observed in a given country. This is necessary to consider to correctly capture the infection dynamics in a country specific manner because in the infection database start time is adjusted to the first infection in the USA. For USA the function $simulation\_start_{Country\_X}$ is turned on at time t = 0 days, but for a county like Austria, where the first reported case was 34 days after first cace in USA, $simulation\_start_{Country\_X}$ was turned on accordingly, considering a incubation time between 2 to 7 days earlier to 34th day; this estimation of $simulation\_start_{Country\_X}$ for Austria was subjected to fitting between a lower and upper boundary of 2 and 7 days respectively assuming a standard incubation time of 5 days.

III. Calculation of of R_0 from the fitted ODE model parameters

If we assume quarantine by testing following the agent based model described in the previous section,

the exposed asymptomatic population would also be quarantined with a rate in addition to the symptomatic infected patients. The equations for this case would be

$$\frac{dS(t)}{dt} = \delta - \delta S(t) - \beta S(t)I(t) - \beta_0 S(t)E(t)$$

$$\frac{dE(t)}{dt} = \beta_0 S(t)E(t) + \beta S(t)I(t) - \alpha_1 E(t) - \alpha_3 E(t) - \delta E(t)$$

$$\frac{dI(t)}{dt} = \alpha_1 E(t) - \alpha_2 I(t) - \delta I(t)$$

$$\frac{dQ(t)}{dt} = \alpha_2 I(t) + \alpha_3 E(t) - \gamma\ Q(t) - \delta Q(t)$$

$$\frac{dR(t)}{dt} = \gamma Q(t) - \delta R(t)$$

In the first equation, the $\beta$and β_0 represent the infection of the susceptible population by infected symptomatic (I) and exposed asymptomatic (E) population. We assumed a natural birth/death rate δ. In the second equation, the exposed individual exhibits symptoms with an incubation rate $\alpha_1$and the exposed population is quarantined with a rate α_3 in order to accommodate quarantine by testing which can also identify asymptomatic exposed individuals. In the third equation, α_2 corresponds to the quarantine of the symptomatic infected people without any requirement for testing. In the fourth equation, the quarantined individual either recovers or dies with rates γ where $\gamma = \gamma_r + \gamma_d$ according to the ODE model described above. The last equation describes the recovery rate. In this calculation, we incorporated both the recovered and death by one equation for R(t).

The jacobian at the infection free equilibrium (I=0,E=0) is given by

$$\begin{bmatrix} -\delta & -\beta_0 & -\beta & 0 \\ 0 & \beta_0-(\alpha_1+\alpha_3+\delta) & \beta & 0 \\ 0 & \alpha_1 & -(\alpha_2+\delta) & 0 \\ 0 & \alpha_3 & \alpha_2 & -(\gamma+\delta) \end{bmatrix}$$

The eigenvalues are

$$\lambda_1 = -\delta, \lambda_2 = -\gamma - \delta$$

$$\lambda_{3,4} = \frac{1}{2}\left(\beta_0 - \alpha_1 - \alpha_3 - 2\delta - \alpha_2\right) \pm \frac{1}{2}\sqrt{\left(\beta_0 - \alpha_1 - \alpha_3 - 2\delta - \alpha_2\right)^2 - 4\left[\left(\beta_0 - \alpha_1 - \alpha_3 - \delta\right)\left(\delta + \alpha_2\right) + \alpha_1\beta\right]}$$

For the infection free equilibrium to be stable

$$\left(\beta_0 - \alpha_1 - \alpha_3 - \delta\right)\left(\delta + \alpha_2\right) + \alpha_1\beta < 0$$

$$R_0 = \frac{\alpha_1\beta}{\left(\alpha_2+\delta\right)\left(\alpha_1+\alpha_3+\delta-\beta_0\right)} < 1$$

This shows that the reproduction ratio R_0 would reduce if quarantine rate is increased by increasing the test rate through α_3. In the original model for fitting, we assumed that α_3 is small and additionally, the natural death rate/birth rate $\delta$is also small in the time scale of the pandemic infection progression so that the total population characteristics remain the same over the timescale. The equation further demonstrates that R_0 would also reduce if the incubation rate $\alpha_1$is high indicating the fact that if the asymptomatic exposed population exhibits symptoms at a higher rate, the spread of infection would be less. However, this will only happen when an asymptomatic carrier is capable of exposing a susceptible person. In fact, if $\beta_0$is negligibly small ($\beta_0 \ll \alpha_3$) in the equation above, the R_0 value would no longer reduce with increasing α_1. This result further illustrates that the effect of asymptomatic spread of infection can be largely mitigated by facilitating more testing and quarantine of the asymptomatic individuals.

According to our calculation shown above, the values of R0 is given by

$$R_0 = \frac{\alpha_1\beta}{(\alpha_2+\delta)(\alpha_1+\alpha_3+\delta-\beta_0)}$$

For the special case we used to fit the data, we assumed that asymptomatic population is not spreading much infection, i.e. $\beta_0 \approx 0$. Additionally, the asymptomatic cases are not quarantined, i.e $\alpha_3 \approx 0$. The δ represents the natural death rate of the population. So the term **δ-δS(t)** corresponds to a birth rate δ and death rate δ for the population where the total population size is normalized to one. Thus at steady state when there is no infection S(t)=1. Now, if we make the final assumption that the natural death rate is small compared to the time scale of infection spread, we can set $\delta = 0$ in the calculation of R0. Consideration of these three assumptions finally produce the R0 as

$$R_0 = \frac{\beta}{\alpha_2}$$

With the introduction of lock down parameter the transmission rate β is replaced as $\beta\frac{\rho}{\rho_{max}}$, finally producing the R0 used in the main text $R_0 = \frac{\beta}{\alpha_2}\frac{\rho}{\rho_{max}}$. Here , $\frac{\rho}{\rho_{max}}$ is the lockdown degree, $\rho = 1$

being the full lockdown case whereas $\rho = \rho_{max}$ being the full unlock scenario. Thus β is the value of transmission rate at full unlock situation.

IV. Model for optimizing the quarantine rate- the cost benefit approach

The basic reproduction ratio for the SEIQR model as derived in Section II is

$R_0 = \frac{\beta}{\alpha_2}\frac{\rho}{\rho_{max}}$ where $\frac{\rho}{\rho_{max}}$ represents the extent of unlocking.

This equation can be transformed as

$$\alpha_2 = \beta\frac{\rho}{\rho_{max}}\frac{1}{R_0}$$

The value of quarantine rate α_2^1 to keep $R_0 = 1$ would be

$$\alpha_2^1 = \beta\frac{\rho}{\rho_{max}}$$

Thus,

$$F_\alpha = \frac{\alpha_2^1}{\alpha_2} = \frac{\left[\beta\frac{\rho}{\rho_{max}}\right]}{\alpha_2}$$

This equation shows that the required fold change F_α in quarantine rate α_2 to the keep $R_0 < 1$ is higher as the extent of lockdown is more. Thus, we argue that the higher F_α has a benefit of unlocking the economy but it comes with a cost in spending financial as well as medical resources in testing. The benefit can be simply written as

benefit$=\frac{F_\alpha}{F_0+F_\alpha}$ takes into account the fact that above certain high quarantine rate F_0 the benefit would saturate. The cost, on the other hand, would also increase with higher quarantine rate as

cost$=\frac{F_\alpha}{F_c-F_\alpha}$ where F_c depicts the maximum capacity of the quarantine rate possible for a particular country on the basis of it's financial and medical resource limitations. The overall balance between the cost and benefit can now be written as

$B = \frac{F_\alpha}{F_0+F_\alpha} - \lambda F_c\frac{F_\alpha}{F_c-F_\alpha}$ where λ represents the resource utilization price per unit of maximum testing rate. At the optimal F_α value, the derivative of the balance equation must be zero. Hence,

$$\frac{\partial B}{\partial F_\alpha} = \frac{F_0}{\left(F_0+F_\alpha\right)^2} - \lambda F_c\frac{F_c}{\left(F_c-F_\alpha\right)^2} = 0$$

$$F_{\alpha}^{opt} = F_c \frac{\left(\lambda \frac{F_c^2}{F_0}\right)^{\frac{3}{2}} + 1}{\left(\lambda \frac{F_c^2}{F_0}\right)^{\frac{1}{2}} + 1}$$

Thus the optimal unlock would be

$$\frac{\beta}{\alpha_2} \frac{\rho_{opt}}{\rho_{max}} = F_c \frac{\left(\lambda \frac{F_c^2}{F_0}\right)^{\frac{3}{2}} + 1}{\left(\lambda \frac{F_c^2}{F_0}\right)^{\frac{1}{2}} + 1}$$

According to the R_0 value from the analytical calculation, it is in general demonstrable that the increased transmission rate can be compensated by increasing the quarantine rate. However, the quarantine rate can be accentuated by increasing the testing rate incurring extra cost. On the other hand, increased transmission rate by partially unlocking the system would benefit the economic transaction.

This can be understood quite intuitively. However, each country has different maximum testing capacity. The maximum testing capacity is defined as the maximum number of tests a country can perform per day. Thus, it is not possible to perform more tests than the maximum due to complete exhaustion of resources. Therefore, the relative cost for tests greater than the maximum would be infinite. At test rate zero, the cost would be zero. The cost would approach infinity at the maximum quarantine rate. The simplest possible function which can achieve both the assumptions that it should be zero at zero quarantine rate ($F_{\alpha} = 0$)and infinite at a maximum quranatine rate ($F_{\alpha} = F_c$) is $\frac{F_{\alpha}}{F_c - F_{\alpha}}$ which is a convex function. From this simple principle, we can argue that the cost curve must be convex as we assumed in the paper based on the assumption that it should monotonically increase with the quarantine rate.

So, the main aim of the cost-benefit model is to quantitatively demonstrate that if the maximum quarantine rate is high, then the relative increase in cost for increasing the quarantine rate by some amount is less compared to the case of increasing the quarantine rate by the same amount when maximum quarantine capacity is low. To demonstrate this we plotted the cost curve for different maximum quarantine rate capacities for a particular country and show that the cost is higher for lower maximum capacity for the same increase in quarantine rate (Figure S4C).

This can also be illustrated by taking derivative of the cost with respect to F_α

$$C = \frac{F_\alpha}{F_c - F_\alpha} \Rightarrow \frac{dC}{dF_\alpha} = \frac{F_c}{(F_c - F_\alpha)^2} \Rightarrow \frac{1}{C}\frac{dC}{dF_\alpha} = \frac{1}{F_\alpha}\frac{F_c}{F_c - F_\alpha}$$

This equation shows that for a constant value of F_α, both the absolute ($\frac{dC}{dF_\alpha}$) and relative changes in cost ($\frac{1}{C}\frac{dC}{dF_\alpha}$) reduce as the maximum capacity F_c increases (since $F_c > F_\alpha$). This further demonstrates that the relative change in cost becomes very high when quarantine rate change F_α approaches the maximum capacity F_c ($F_c \approx F_\alpha$) owing to exhaustion of resources. This conclusion would remain the same irrespective of the existence of optimum.

As long as the benefit is monotonically increasing with the lockdown opening, at least one maximum would always exist in the cost-benefit trade-off. However, if benefit is so low that the benefit curve never intersects the cost curve, there will be no optimum giving rise to a hopeless situation where no amount of testing is sufficient to open the lockdown.

V. A case for the periodic lockdown

In a previous study, it was suggested that a periodic lockdown may be a good strategy to optimize unlock and economic activity [1]. Thus, if after every T_1 time of full unlock, a lockdown period is imposed for a period of T_2 over a total time N and this cycle continues. Hene, over a cycle of period T, for the interval T_1 , the $\frac{\rho}{\rho_{max}} = 1$and followed by an interval T_2 where $\rho = 1$. In this case one can show that in the allowed unlock period the average R_0 would be given by

$$\langle R_0 \rangle = \frac{\alpha_1 \beta}{\alpha_2 (\alpha_1 + \alpha_3)} \frac{1}{\rho_{max}} \left[\left(\rho_{max} - 1 \right) \frac{T_1}{T} + 1 \right]$$

(here α_3 corresponds to the quarantine rate through testing in addition to quarantine rate α_2 of only symptomatic individuals). Equation above shows that the reproduction number during lockdown ($\frac{\alpha_1 \beta}{\alpha_2 (\alpha_1 + \alpha_3)} \frac{1}{\rho_{max}}$) must be less than one to maintain the average R_0 less than one. This shows that as the effect of lockdown ρ is high the allowed unlock period would also be high. The allowed period can

further be stretched by increasing the quarantine rate involved with testing and quarantining the exposed subjects at a rate α_3 where as small value of α_3 corresponds to the case when testing rate is low which is used in fitting the data.

Frequency of the unlock can be connected to the extent of unlock through the equation below

$$\frac{T_1}{T} = \frac{\left(\frac{\rho}{\rho_{max}} - \frac{1}{\rho_{max}}\right)}{\left(1 - \frac{1}{\rho_{max}}\right)}$$

This quantifies the frequency of unlock required within a time N to implement a particular extent of overall unlock $\frac{\rho}{\rho_{max}}$ (Figure S4A). The corresponding unlock period to keep the average value at 1 is given by

$$\frac{T_1}{T} = \frac{\frac{\alpha_2(\alpha_1+\alpha_3)}{\alpha_1\beta} - \frac{1}{\rho_{max}}}{1 - \frac{1}{\rho_{max}}} \qquad (6)$$

which represents allowed number of unlock days over a period time T to keep the average R_0 at equal to 1. Hence, as the effect of lockdown ρ_{max} is high the allowed unlock interval can also be longer. The equation further reveals that the allowed period of working days (T_1 can further be stretched by increasing the quarantine rate (α_3) (Figure S4B) involved with testing and quarantining the exposed subjects whereas small value of α_3 corresponds to the case when the testing rate is low assumed in fitting the data.

VI. Scaling relation between agent based model and analytical model

In order to investigate the scaling between ODE based and agent based models, we calculated the quarantine rate ($\alpha_2^1 = F_\alpha \alpha_2$, where α_2 is the fitted country specific quarantine rate) required to maintain R_0 at a value of 1 from the analytical calculation (equation (1) in main text) and the corresponding $10000 \times N_{test}/(N_T T)$ (N_T=Number of test per 10000 per day where $N_T = 300 \times 300,\ T = 200\ days$) value required to maintain R_0 at 1 from the agent based model. We then performed the calculation for all the countries at different unlock degrees and plotted them (Figure S6G). So, the x-axis displays the testing rate required to keep R0<1 determined by the ABM whereas

the y-axis represents the quarantine rate required to keep R0<1 determined from the analytical calculation. For example, the four red points correspond to four unlock degrees for the USA (square : Full unlock, diamond: 75 % unlock, triangle: 57% unlock and circle: 37% unlock). Similar procedures were followed for other countries as indicated in the figure. Indeed, we found that all points fall on a straight line displaying a high correlation. This result suggests that the ODE-based model and agent-based model scale in a linear fashion with each other, if we use the same parameter values for both cases and keep the population size the same for all the countries. Thus, one can convert fold change in quarantine rate to fold change in testing rate with a linear scaling.

VII. References

1. Omer Karin *et al.* (2020) Adaptive cyclic exit strategies from lockdown to suppress COVID-19 and allow economic activity. ***medRxiv*** doi: https://doi.org/10.1101/2020.04.04.2005357
2. Raue A, Steiert B, Schelker M, Kreutz C, Maiwald T, Hass H, Vanlier J, Tönsing C, Adlung L, Engesser R, Mader W, Heinemann T, Hasenauer J, Schilling M, Höfer T, Klipp E, Theis F, Klingmüller U, Schöberl B, Timmer J (2015) Data2Dynamics: a modeling environment tailored to parameter estimation in dynamical systems. Bioinformatics 31: 3558–3560. doi: 10.1093/bioinformatics/btv405.

VIII. Supplementary Figure legends

Figure S1: (A) The regions highlighted in figure 1B are expanded to display the country names (B) The correlation between test rate and doubling rate at two different time points is shown using scatter plot (t=6 and 30 corresponds to the time after alignment as described in the previous section) (C) The scatter plot between test rate and daily confirmed cases for generating the Figure 1C and 1D is shown at two different time points, as an example. (D) normalized daily cases are shown and then these three time traces are aligned at the maximum doubling rate. On the left panel, the dashed vertical line at zero in the doubling rate aligned figure corresponds to the maximum doubling rate time point for each country. Right panel displays the original confirmed cases before alignment.

Figure S2: The SEIQR models fit the data for 50 countries, as indicated, for the cumulative confirmed cases. The figures indicate the cumulative data and the corresponding fit is based on the SEIQR model

explained the study.The number of days in X axis corresponds to the time course data available in JHU CSSE [40] where 0 coropospents to 22nd January, 2020 and the end time point corresponds to 15th August, 2020. It can be noted that keeping the fitted parameters constant but varying extent of lockdown the future increase or decrease in the infection spread trajectory for a given country can be estimated.

Figure S3: (A)The scatter plot showing the relationship between the R_0 and different demographic and medical facility factors as indicated in the figures and in their legend. (B) The scatter plot shows the relationship between the frequencies of seven clades in different countries and corresponding R_0 values.

Figure S4: (A) The implementation of the extent of unlock as a function of the periodic unlock for the different countries are indicated. Number of working days represents the working day cycle in 15 days time. (B) The quarantine required to be increased by testing to keep the R_0 value below one and the corresponding the working days allowed. (C) The cost curve for different maximum quarantine rate capacities as indicated for a particular country.

Figure S5: The dynamics of the spread of the infection starting from the initial one infected agent at the centre is shown as a representative output of the agent based stochastic simulation. This example is the result of a simulation for parameter values of the USA with full unlock condition at the testing rate 2.4/10000. The blue, red, green and cyan respectively represent susceptible, undetected infected(asymptomatic+symptomatic), quarantined and recovered agents respectively. The quarantine is performed by testing individuals through contact tracing of the already quarantined patients.

Figure S6: (A) The time traces of the confirmed daily cases of agent based simulation based on parameter values with full unlock for the USA at different daily tests/10000 as indicated. The points indicate the values obtained from the simulations; the solid lines represent LOESS regression fits. (B-E) The relationship between the R_0 values and the testing rates for different extents of unlock are shown, as indicated, for different countries. (F) The total number of confirmed cases evolving with time is shown for different percentages of superspreaders, shown here for a testing rate of 9/10000. (G) The plot shows the scaling relation between the ABM and analytical calculation. The x-axis displays

the testing rate required to keep R0<1 determined by the ABM whereas the y-axis represents the quarantine rate required to keep R0<1 determined from the analytical calculation.
